# Association Between Red and Processed Meat Consumption and Risk of Prostate Cancer: A Systematic Review and Meta-Analysis

**DOI:** 10.3389/fnut.2022.801722

**Published:** 2022-02-07

**Authors:** Saeedeh Nouri-Majd, Asma Salari-Moghaddam, Azadeh Aminianfar, Bagher Larijani, Ahmad Esmaillzadeh

**Affiliations:** ^1^Department of Community Nutrition, School of Nutritional Sciences and Dietetics, Tehran University of Medical Sciences, Tehran, Iran; ^2^Research Center for Biochemistry and Nutrition in Metabolic Diseases, Kashan University of Medical Sciences, Kashan, Iran; ^3^Obesity and Eating Habits Research Center, Endocrinology and Metabolism Molecular-Cellular Sciences Institute, Tehran University of Medical Sciences, Tehran, Iran; ^4^Department of Community Nutrition, School of Nutrition and Food Science, Isfahan University of Medical Sciences, Isfahan, Iran

**Keywords:** red meat, processed meat, total meat, prostate cancer, meta-analysis

## Abstract

**Background:**

Debate on the potential carcinogenic effects of meat intake is open and the relationship between meat consumption and risk of prostate cancer remains uncertain. This meta-analysis was conducted to summarize earlier prospective studies on the association of meat consumption with risk of prostate cancer.

**Methods:**

Relevant studies were identified by exploring PubMed/Medline, Scopus, Web of Science, EMBASE, and Google Scholar databases up to December 2020. Fixed-effects and random-effects meta-analyses were used for pooling the relative risks (RRs). Heterogeneity across studies was evaluated using the Q-statistic and *I*-square (*I*^2^). A funnel plot and Egger's test was used to detect publication bias. Linear and non-linear dose-response analyses were performed to estimate the dose-response relations between meat intake and risk of prostate cancer.

**Results:**

Twenty-five prospective studies were included in this meta-analysis. Totally, 1,900,910 participants with 35,326 incident cases of prostate cancer were investigated. Pooling the eligible effect sizes, we observed that high consumption of processed meat might be associated with an increased risk of “total prostate cancer” (RR: 1.06; 95% CI: 1.01, 1.10; *I*^2^ = 1.5%, *P* = 0.43) and “advanced prostate cancer” (1.17; 1.09, 1.26; *I*^2^ = 58.8%, *P* = 0.01). However, the association between processed meat and “advanced prostate cancer” was not significant in the random-effects model: 1.12 (95% CI: 0.98, 1.29). A linear dose-response analysis indicated that an increment of 50 grams per day of processed meat intake might be related to a 4% greater risk of “total prostate cancer” (1.04; 1.00, 1.08; *I*^2^ = 0.0%, *P* = 0.51). “Total meat intake” was marginally associated with all outcomes of prostate cancer risk (1.04; 1.01, 1.07; *I*^2^ = 58.4%, *P* < 0.001).

**Conclusions:**

This systematic review and meta-analysis of prospective studies indicated that increased consumption of “total meat” and “processed meat” might be associated with a higher risk of prostate cancer.

**Systematic Review Registration:**

https://www.crd.york.ac.uk/prospero/display_record.php?RecordID=230824, identifier: CRD42021230824.

## Introduction

Prostate cancer is the second most frequent cancer and the fifth leading cause of cancer death among men worldwide (1, 2). It is the most commonly diagnosed cancer in 12 regions of the world in men with an incidence rate of 13.5%, globally (2).

Older age, African-American descent, and family history are the established risk factors for prostate cancer. Diet is one of the most modifiable risk factors for prostate cancer (3). Consumption of some food groups has been positively or inversely associated with the risk of prostate cancer (4, 5). The most interesting food group in this regard is the consumption of meat and meat products. The relation between meat intake and risk of prostate cancer has been widely investigated; however, findings are controversial (6, 7). Red and processed meat contain heme iron and other compounds including heterocyclic amines (HCAs), polycyclic aromatic hydrocarbons (PAHs), and N-nitroso compounds (NOCs) that are produced by high-temperature or prolonged cooking (8, 9). These compounds were reported to be carcinogenic in animal studies (10). Earlier studies have shown a positive association between red and processed meat consumption with prostate cancer risk (6, 11). However, previous two meta-analyses of prospective cohort studies did not find a relationship between red or processed meat consumption and risk of developing prostate cancer (12, 13), except for a weak positive association between processed meat intake and total prostate cancer risk (13). In a pooled analysis of 15 cohort studies in 2016, total red meat, unprocessed red meat, and processed meat consumption were not associated with risk of all prostate cancer (14). Five new big cohort studies were published since the release of the last meta-analysis. Furthermore, no previous study had examined the non-linear dose-response association between meat consumption and risk of prostate cancer. In the current study, we did an updated systematic review and a comprehensive dose-response meta-analysis of previous studies on the relationship between red and processed meat consumption and risk of prostate cancer.

## Methods And Materials

### Search Strategy

This systematic review and meta-analysis presented based on PRISMA guideline (15). The protocol for this review was registered at PROSPERO (registration no. CRD42021230824). We investigated the electronic databases of PubMed/Medline, Scopus, Web of Science, EMBASE, and Google Scholar systematically to find relevant studies. In this search, we used keywords including the following terms: (“prostatic neoplasms” OR “prostate cancer” OR “prostatic neoplasms” OR “prostate”) AND (“red meat” OR “meat” OR “meat products” OR “pork meat” OR “meat” OR “meat products” OR “red meat” OR “minced meat” OR “beef” OR “mutton” OR “pork” OR “veal” OR “lamb” OR “processed meat” OR “hamburger” OR “salami” OR “hot dog” OR “bacon” OR “sausage”). No restriction was used when searching the databases. To avoid missing any relevant study, we reviewed the reference lists of all related publications. Duplicate citations were then removed. All potentially relevant studies identified from the literature search were screened by two independent investigators (SNM and AA) based on the study title and abstract. Any disagreements were resolved in consultation with the principal investigator (AE).

### Inclusion Criteria

Studies were included in this systematic review and meta-analysis if they met the following criteria: (1) publications done on men > 18 years old; (2) those that assessed consumption of red or processed meat as the exposure; (3) examined high vs. low meat consumption; (4) investigated risk of prostate cancer as the outcome; and (5) nested case-control, case-cohort, and prospective cohort studies.

### Exclusion Criteria

We excluded duplicate citations and those that did not meet our inclusion criteria. Studies that assessed consumption of meat, chicken, or fish intake together were excluded (some studies that considered white meat as part of the processed meat were not excluded). We also did not include studies that investigated the incident symptomatic benign prostatic hyperplasia as the outcome. In addition, case-control, cross-sectional, ecological design, reviews, editorials, commentaries, and letters were not included as well.

### Data Extraction

The following information was extracted with a standardized data collection form by two reviewers (SNM and AA): the first author's last name, name of the study cohort, country, participants' age (mean or range), number of participants, number of cases, years of follow up (mean, median, or maximum number of follow up), method of assessment of meat intake, the main exposure and outcome of interest, comparisons, the relevant effect size [including odds ratios (ORs), risk ratios or relative risks (RRs), and hazard ratios (HRs)] and 95% confidence intervals (CIs), and covariates used for adjustment. Any disagreements in data extraction between the two reviewers were consulted with the leading investigator (AE).

“Red meat” was defined as the consumption of red meat and unprocessed red meat. “Processed meat” was defined as the consumption of sausages, bacon, hamburger, ham, lunch meat, hot dogs, cured meat, cold meat, smoked beef, and processed poultry, poultry sausage. “Red and processed meat” was considered as the sum of red meat and processed meat. In addition, “total meat” was defined as the sum of red meat, processed meat, and meat (in publications where meat consumption had not been defined to be red or white meat). The studies had defined the outcome in different ways. In the current meta-analysis, studies with the outcome of prostate cancer, total prostate cancer, all prostate cancer, and prostate cancer diagnosis were included in the category of “total prostate cancer.” In addition, “advanced prostate cancer” in this study was defined as advanced prostate cancer, high-stage prostate cancer, lethal prostate cancer, fatal prostate cancer, non-localized or high-grade cancer, and metastatic prostate cancer.

### Excluded Articles

**Figure 1:**
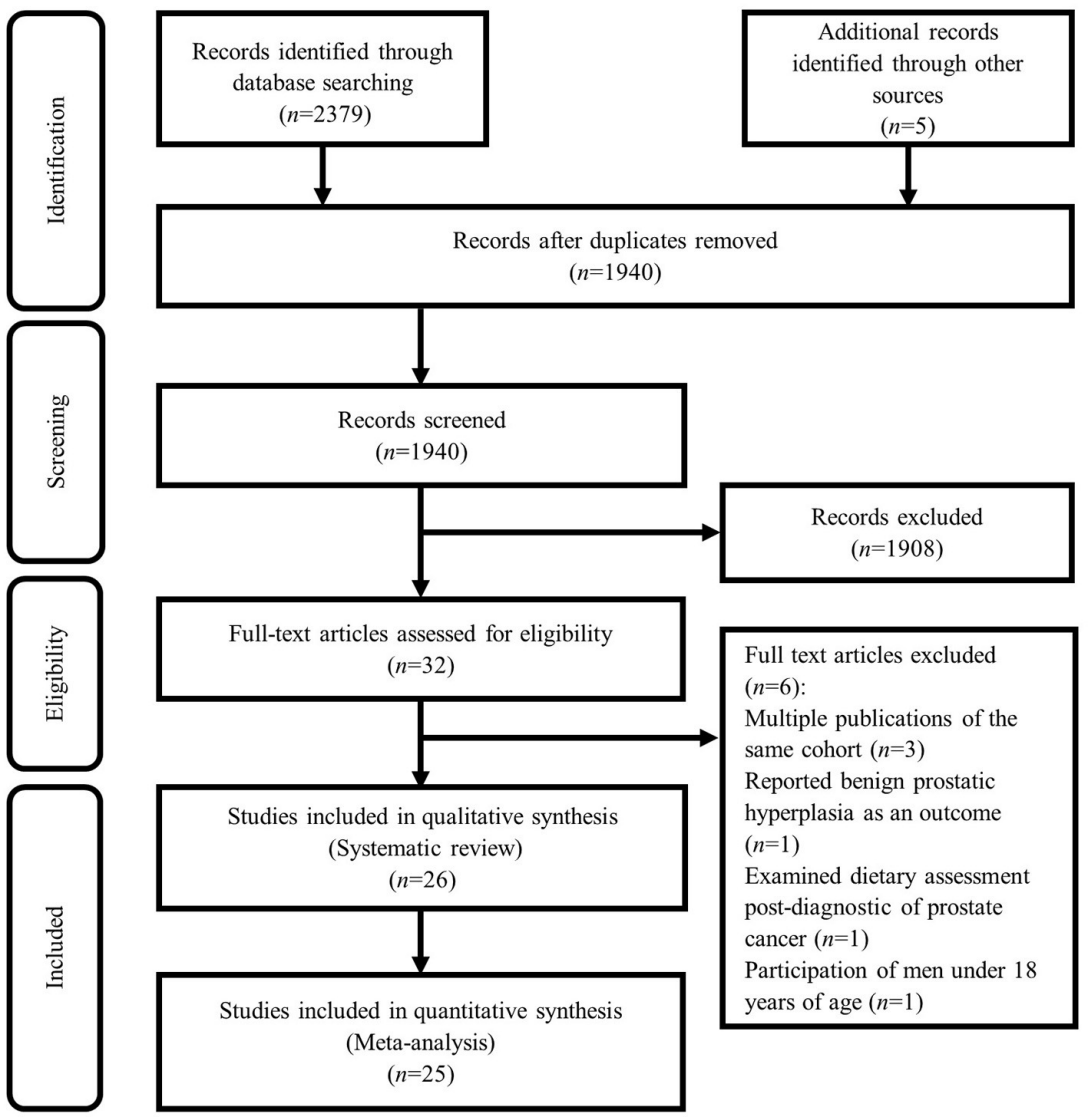
Flowchart of the study selection process.

Based on our initial search, 1,940 studies were found. Based on screening for title and abstract, a total of 1,908 articles were excluded and 32 articles remained to be assessed for eligibility. After evaluation, 6 further studies were excluded due to the following reasons: three cohort studies with the same population in other publications including NIH-AAPR cohort (16, 17), and ATBC cohort (18), in which we considered the most comprehensive publication (19). In other words, among studies published from the NIH-AAPR cohort, we included the study of Sinha et al. and excluded the studies of Major et al. and Cross et al. because the study of Sinha et al. had considered a larger sample size. Also, among studies published from the ATBC cohort, we included the study of Wright et al. because had considered a more follow-up duration. The study of Kristal et al. was excluded because of considering incident symptomatic benign prostatic hyperplasia as the outcome, rather than prostate cancer (20). In addition, we excluded the study of Richman et al. because of considering post-diagnostic dietary intakes (21). Also, the study of Veierød et al. was excluded due to the participation of men under 18 years of age (22). Therefore, a total of 26 studies remained for the current systematic review. In the meta-analysis, we included 25 (6, 7, 11, 23–44), out of these 26 studies, because the study of Orenstein et al. did not report the required effect sizes (45). The details of the study selection process are shown in Figure 1.

### Quality Assessment of Studies

The quality of each study was assessed using the Newcastle-Ottawa Scale (NOS). To ensure that the scoring of studies is unbiased, scoring was done by two independent investigators (SNM and AA). This scale includes three parameters for quality assessment: selection, comparability, and outcomes for cohort study. Each study can receive a maximum of four stars for selection, two stars for comparability, and three stars for the outcome. Therefore, in total, each study can achieve a maximum of 9 stars (46). We defined studies with NOS scores of ≥7 as high-quality studies and those with a score of <7 as low-quality studies.

### Statistical Analysis

In this meta-analysis, we included ORs, RRs, and HRs for the nested case-control, case-cohort, and prospective cohort studies. Given that random-effects meta-analysis might result in some bias for small studies (47, 48), we applied a fixed-effects model to compute RRs estimates and 95% CIs in this analysis. However, random-effects model was also applied. Q-statistic and *I*-square (*I*^2^) were used to evaluate heterogeneity across the studies. Significant heterogeneity between studies was indicated if *I*^2^ > 50%. Subgroup analyses were used to find the possible sources of heterogeneity. Between-study heterogeneity was assessed using a fixed-effects model. These analyses were done based on predefined criteria, including country, study quality, and adjustment for energy intake, smoking, alcohol consumption, and family history of cancer. For one study (26) that reported risk estimates for the lowest vs. highest categories of processed meat intake, the risk estimates were computed for the highest vs. the lowest categories of processed meat intake using the Orsini method (49). We conducted a sensitivity analysis to evaluate the influence of a single study on the overall meta-analysis estimate. The possibility of publication bias among included studies was examined by visual evaluation of a funnel plot and the Egger's test. For the egger's test, *P* values < 0.10 were considered as statistically significant. If there was a significant publication bias, we examined the influence of a publication bias on the findings using the “trim and fill” method (50). A random-effects linear dose-response meta-analysis was performed to estimate the pooled RRs and 95% CIs of prostate cancer for each additional 50 g/day red and processed meat consumption. To do this, the generalized least squares trend estimation method was used, as suggested by Orsini et al., and Greenland and Longnecker (49, 51). Primarily, study-specific slope lines were estimated, followed by combining these lines to obtain an overall average slope. In the dose-response analysis, if the total number of participants or cases in each category was not reported, we divided the total number by the number of categories (52). The median or mean amount of meat intake in each category was allocated to the corresponding RR for each study. For studies that stated the intake as ranges, we estimated the midpoint in each category by calculating the mean of the lower and upper bound. If the highest category was open-ended, the length of the open-ended interval was assumed to be the same as that of the adjacent interval. For studies that reported meat intake as serving or time, we considered 120 grams of red meat, 50 grams of processed meat, and 85 grams of total red and processed meat as a serving, as used in previous studies (53). For studies that stated grams per 1,000 kcal, we calculated the reported intakes using the mean energy intake or 2,000 kcal daily intake. We also examined the non-linear dose-response association between meat intake and prostate cancer risk. Meat consumption was modeled by using restricted cubic splines with 3 knots at percentiles of 10, 50, and 90% of the distribution. The correlation within reported risk estimates was considered and the study-specific RRs were combined using a one stage linear mixed effects meta-analysis. Considering the null hypothesis testing, the significance for non-linearity was computed assuming the coefficient of the second spline equal to zero. Statistical analyses were performed using STATA version 14. *P* values < 0.05 were considered statistically significant.

## Results

### Study Characteristics

Characteristics of included studies are provided in Supplementary Table 1. Sixteen studies were reported from the United States (US) (6, 11, 26, 28, 31–36, 38, 40–44), seven from Europe (7, 23, 25, 27, 29, 30, 39), and two from East Asia (24, 37). The number of participants in these studies ranged from 240 to 1,179,172. Totally, 1,900,910 participants with 35,326 incident cases of prostate cancer were investigated in these publications. Participants were followed up for 6 to 23 years. Participants aged over 18 years old. Assessment of meat consumption was mostly done using a food frequency questionnaire (FFQ), except for three studies that used dietary records (7, 23, 25), and four studies that used an unidentified questionnaire (24, 31, 40, 42). Most studies had controlled for age (*n* = 23), smoking (*n* = 14), energy intake (*n* = 13), body mass index (BMI) (*n* = 13), family history of cancer (*n* = 9), and education (*n* = 7). Out of 25 studies, 11 studies were of high quality (NOS ≥ 7) (7, 11, 23, 25, 27, 28, 34–36, 38, 44) and other articles were of low quality (NOS < 7, *n* = 14) (6, 24, 26, 29–33, 37, 39–43). For studies that reported the effect sizes for different types of red or processed meat separately (31, 34, 40, 43), we combined the effect sizes using a fixed-effects model and then included the final effect size in the meta-analysis. This was also the case for studies that had reported effect sizes separately for different age (35) or race groups (11).

Considering red meat consumption as the exposure, we found that four studies reported a positive association with risk of total prostate cancers (6, 23, 29, 40). However, in one study, a significant relationship was observed only with beef (40). No significant relationship was found in the remaining 12 studies (7, 11, 25, 27, 30, 31, 33, 34, 36, 37, 41, 45). In terms of advanced prostate cancer, one study found a significant positive association with red meat intake (6), but there was no significant relationship in 8 studies (11, 27, 29, 31, 33–36). About processed meat consumption, a significant association was found between processed meat intake and total prostate cancer in four studies (6, 11, 34, 39). However, in one study this association was seen only in African Americans (11). In another study, it was significant for ham/lunch meat intake (34). No significant relationship was observed in 10 studies (7, 23, 25, 27, 30, 31, 33, 36, 40, 44). Three studies found a significant association between processed meat consumption and advanced prostate cancer (6, 26, 34). Among these studies, one study found this association only for sausage intake (34). Five studies reached no significant association between processed meat intake and risk of advanced prostate cancer (11, 27, 31, 33, 36).

### Findings From the Meta-Analysis

Twenty-five articles were included in this meta-analysis. We performed the analysis based on red meat, processed meat, red and processed meat, and total meat (all meat, red and processed meat) consumption separately. In addition, in each group, the analyses were done separately for total prostate cancer and advanced prostate cancer. However, a pooled analysis for all outcomes of prostate cancer was also performed.

### Meta-Analysis on “Red Meat Intake” and Risk of “Total Prostate Cancer”

**Figure 2:**
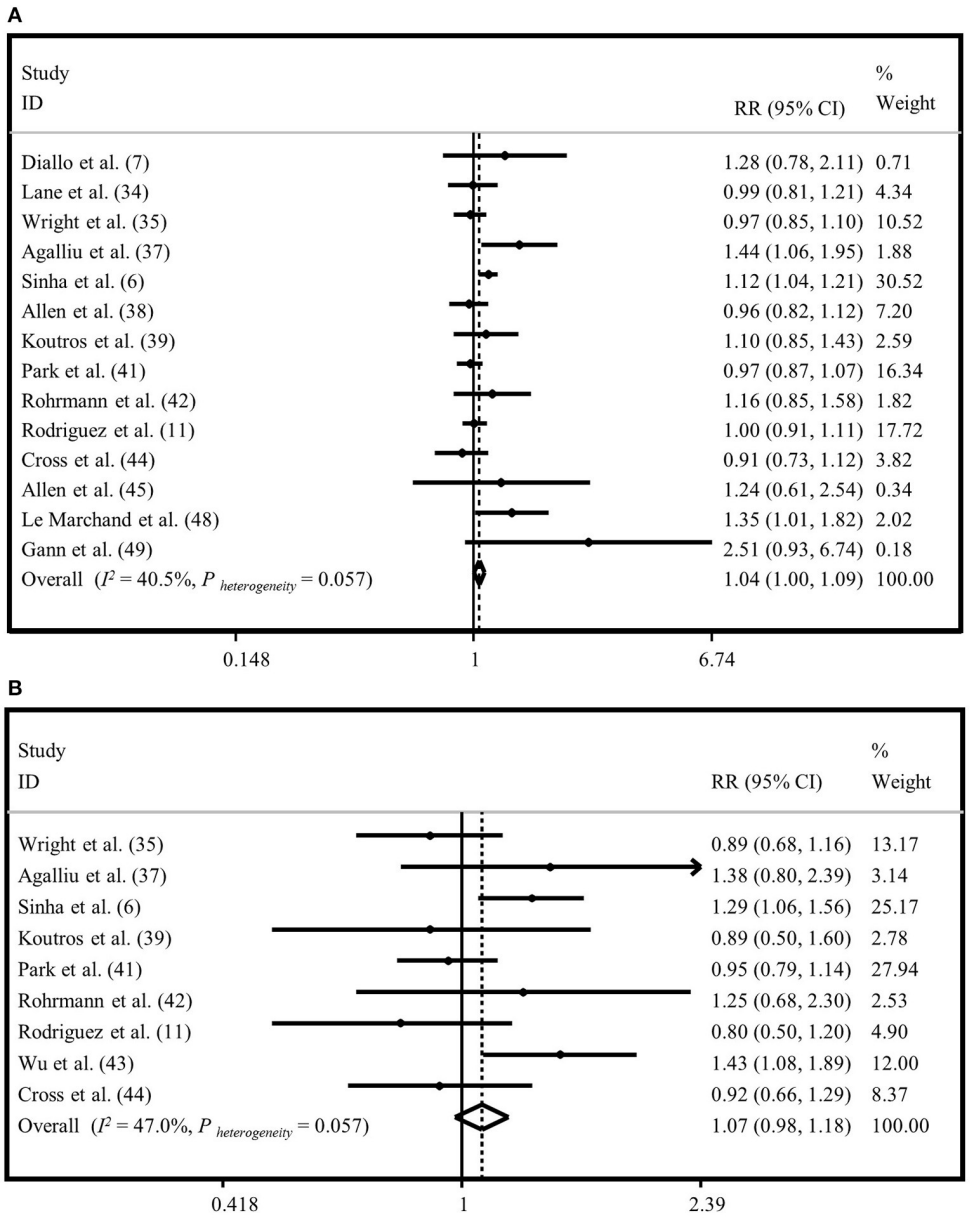
Forest plot derived from fixed-effects meta-analysis investigating the association between red meat intake and risk of total prostate cancer **(A)** and advanced prostate cancer **(B)**. RR, relative risk; CI, confidence intervals; I^2^, I-square.

Fourteen studies assessed the association between red meat intake and risk of total prostate cancer (6, 7, 11, 25, 27, 29–31, 33, 34, 36, 37, 40, 41). The summary risk estimate based on fixed-effects model for “total prostate cancer,” comparing the highest vs. lowest “red meat intake” was 1.04 (95% CI: 1.00, 1.09) (Figure 2A). When we applied random-effects model, this finding became non-significant (1.05; 95% CI: 0.98, 1.12) (Supplementary Figure 1A). Heterogeneity was moderate (*I*^2^ = 40.5%, *P* = 0.05). There was no evidence of publication bias (Egger's test *P* = 0.173). The results from the subgroup analyses revealed that adjustment for energy intake, alcohol consumption, and family history of cancer had influenced the association of “red meat intake” and risk of “total prostate cancer” (Supplementary Table 2).

### Meta-Analysis on “Red Meat Intake” and “Advanced Prostate Cancer”

Totally, nine publications examined the association between consumption of “red meat” and risk of “advanced prostate cancer” (6, 11, 27, 29, 31, 33–36). Comparing extreme categories, no significant association was found between “red meat intake” and risk of “advanced prostate cancer” based on fixed-effects model (RR = 1.07, 95% CI: 0.98, 1.18) (Figure 2B). The same findings were obtained when we applied random-effects meta-analysis (RR = 1.07, 95% CI: 0.92, 1.24) (Supplementary Figure 1B). The results of heterogeneity and publication bias analysis revealed no significant between-study heterogeneity (*I*^2^ = 47.0%, *P* = 0.05) and no evidence of publication bias (Egger's test *P* = 0.857). Findings from subgroup analyses are provided in Supplementary Table 2.

### Meta-Analysis on “Processed Meat Intake” and “Total Prostate Cancer”

**Figure 3:**
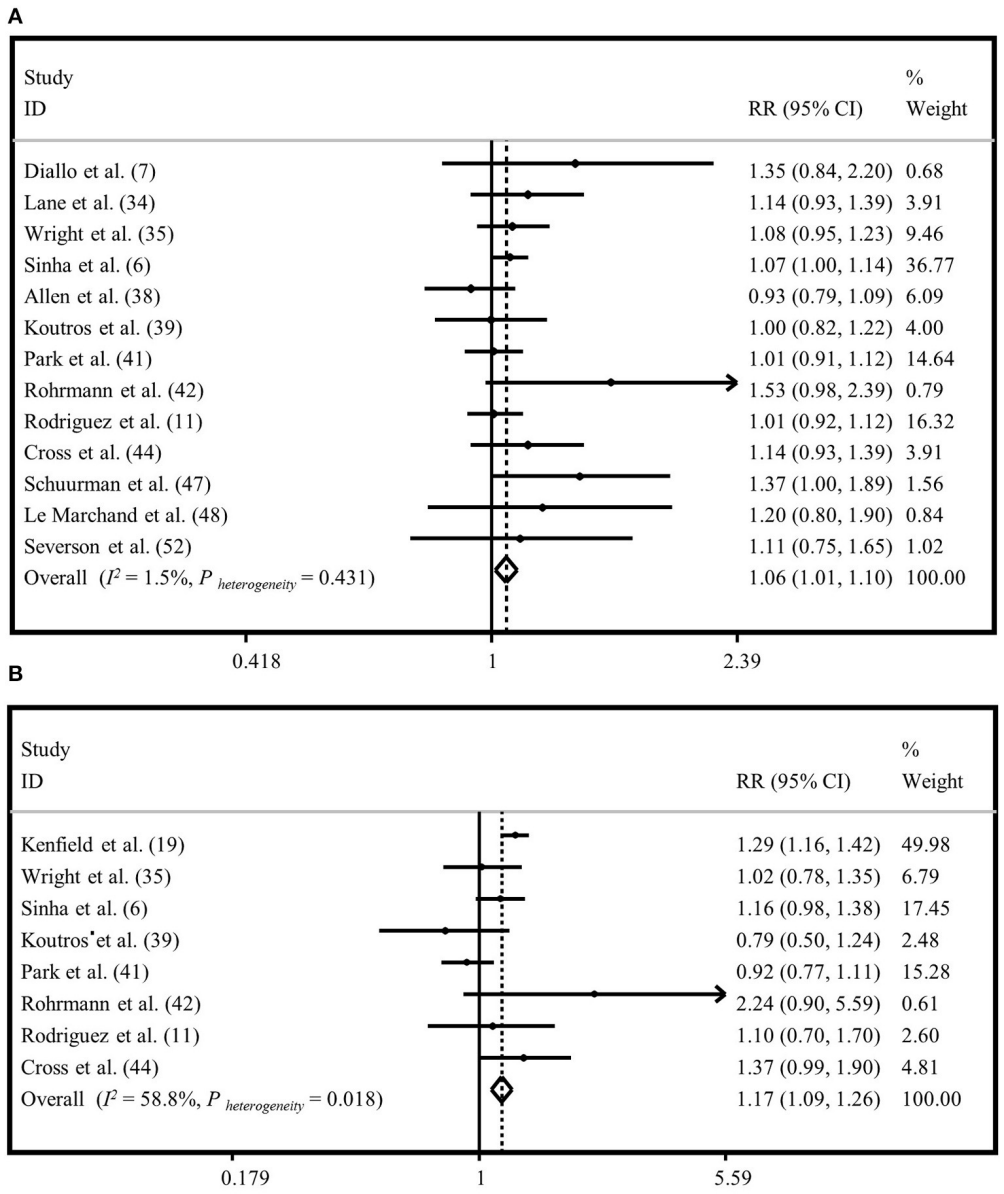
Forest plot derived from fixed-effects meta-analysis investigating the association between processed meat intake and risk of total prostate cancer **(A)** and advanced prostate cancer **(B)**. RR, relative risk; CI, confidence intervals; I^2^, I-square.

To investigate the association between “processed meat intake” and risk of “total prostate cancer,” 13 studies were included (6, 7, 11, 25, 27, 30, 31, 33, 34, 36, 39, 40, 44). A significant relationship was observed when the highest category of “processed meat intake” was compared to the lowest intake based on both random and fixed-effects analyses (RR = 1.06, 95% CI: 1.01, 1.10). No evidence of heterogeneity was seen between studies (*I*^2^ = 1.5%, *P* = 0.43) (Figure 3A; Supplementary Figure 2A). Publication bias was seen by Egger's test (*P* = 0.06). The influence of a publication bias on the findings was examined using the ‘trim and fill' analysis. After imputing four hypothetically missing effect sizes in this analysis, the results were still statistically significant in the fixed-effects model (RR = 1.04, 95% CI: 1.00, 1.08), but not in the random-effects model (1.04; 95% CI: 0.99, 1.09). Findings from subgroup analyses are provided in Supplementary Table 2.

### Meta-Analysis on “Processed Meat Intake” and “Advanced Prostate Cancer”

Eight publications were included to assess the association between “processed meat intake” and the risk of “advanced prostate cancer” (6, 11, 26, 27, 31, 33, 34, 36). High intake of “processed meat” was positively associated with the risk of “advanced prostate cancer” in the fixed-effects model (RR=1.17, 95% CI: 1.09, 1.26), with a moderate heterogeneity between studies (*I*^2^ = 58.8%, *P* = 0.01) (Figure 3B). However, there was no significant association between “processed meat intake” and the risk of “advanced prostate cancer” in the random-effects model (RR = 1.12, 95% CI: 0.98, 1.29) (Supplementary Figure 2B). Egger's regression test revealed no statistical evidence of publication bias (*P* = 0.569). Subgroup analyses were conducted to find the sources of between-study heterogeneity (Supplementary Table 2). Subgroup analyses revealed that adjustment for energy intake and family history of cancer might provide some reasons for between-study heterogeneity.

### Meta-Analysis on “Red and Processed Meat Intake” and “Total Prostate Cancer”

The relationship between “red and processed meat intake” and “total prostate cancer” risk was investigated using thirteen studies (6, 7, 11, 25, 27, 30, 31, 33, 34, 36, 38, 40, 43). The association was not significant between “red and processed meat intake” and risk of “total prostate cancer,” comparing the highest and lowest categories in the fixed-effects model (RR = 1.02, 95% CI: 0.99, 1.05) (Supplementary Figure 3A) and random-effects model (RR = 1.01, 95% CI: 0.96, 1.05) (Supplementary Figure 4A). Heterogeneity was not significant between studies (*I*^2^ = 42.1%, *P* = 0.05). We found no evidence of publication bias among the included studies (Egger's test *P* = 0.551). Based on subgroup analyses, we found that country and adjustment for alcohol consumption might explain between-study heterogeneity (Supplementary Table 2).

### Meta-Analysis on “Red and Processed Meat Intake” and “Advanced Prostate Cancer”

Nine studies were included to investigate the relationship between “red and processed meat intake” and risk of “advanced prostate cancer” (6, 11, 27, 31, 33–36, 42). The summary risk estimate for “advanced prostate cancer,” comparing the highest and lowest categories of “red and processed meat intake,” was 1.05 (95% CI: 0.97, 1.12) in the fixed-effects model (Supplementary Figure 3B), and 1.00 (95% CI: 0.87, 1.15) in the random-effects analysis (Supplementary Figure 4B), indicating that there was no significant association between “total red and processed meat intake” and risk of “advanced prostate cancer.” Heterogeneity between studies was 63.3% (*P* = 0.005). No evidence of publication bias was observed by Egger's test (*P* = 0.292). We performed subgroup analyses to assess sources of between-study heterogeneity. In the subgroup analyses, we found that country and adjustment for alcohol consumption might describe between-study heterogeneity (Supplementary Table 2).

### Meta-Analysis on “Total Meat Intake” and “Total Prostate Cancer”

**Figure 4:**
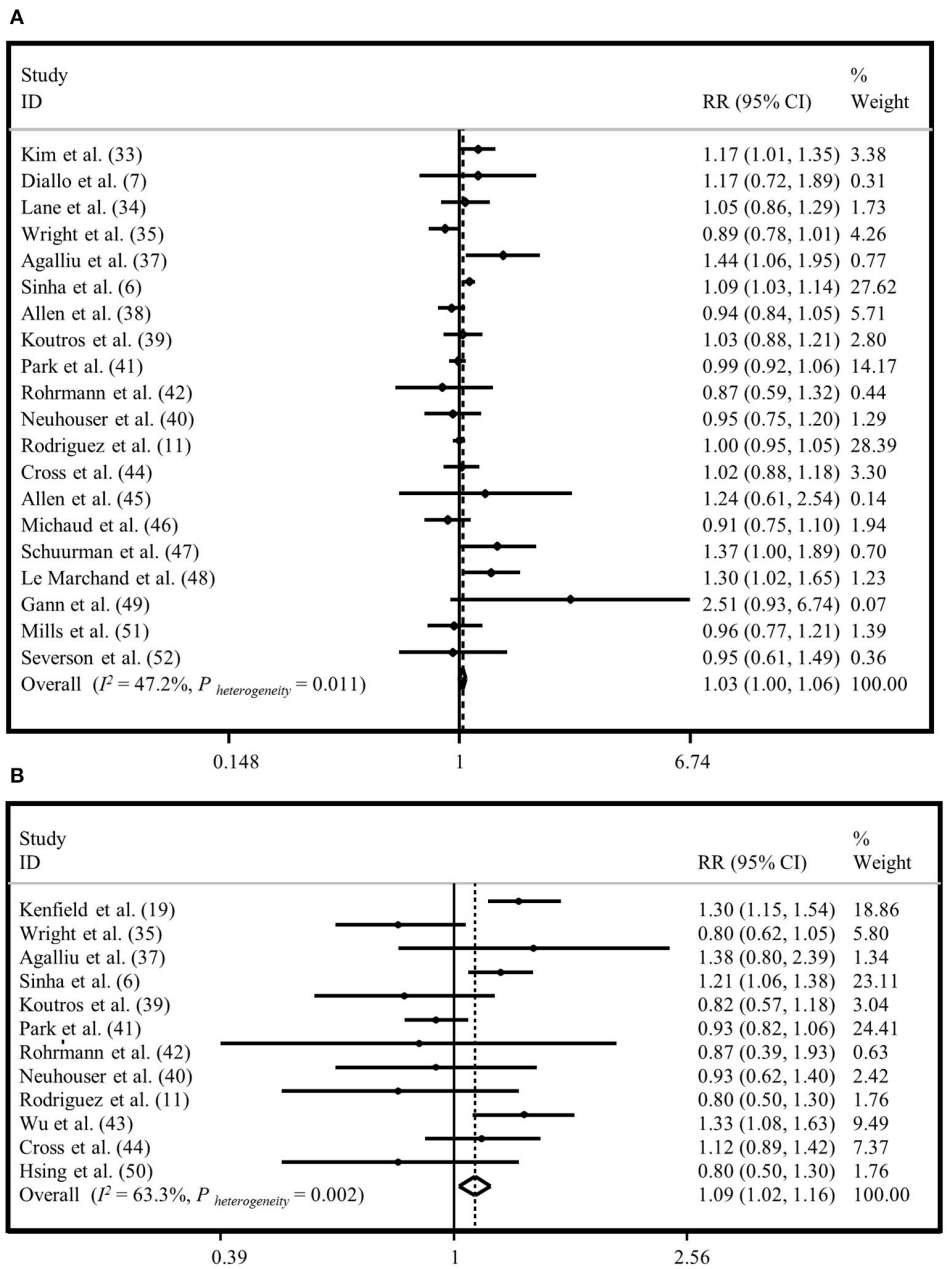
Forest plot derived from fixed-effects meta-analysis investigating the association between total meat intake and risk of total prostate cancer **(A)** and advanced prostate cancer **(B)**. RR, relative risk; CI, confidence intervals; I^2^, I-square.

Twenty studies were used to evaluate the association between “total meat intake” (total meat, meat, red meat, processed meat, and red and processed meat intake) and risk of “total prostate cancer” (6, 7, 11, 24, 25, 27, 29–34, 36–41, 43, 44). We found a marginal positive relationship between “total meat intake” and risk of “total prostate cancer” in the fixed-effects model (1.03; 95% CI: 1.00, 1.06; *I*^2^ = 42.2%, *P* = 0.01) (Figure 4A), but not in the random-effects analysis (RR=1.03, 95% CI: 0.98, 1.08) (Supplementary Figure 5A). Results of Egger's test indicated no publication bias (Egger's test = 0.413). Based on subgroup analyses, adjustment for energy intake and alcohol consumption, and study quality score might be potential sources of heterogeneity (Supplementary Table 2).

### Meta-Analysis on “Total Meat Intake” and “Advanced Prostate Cancer”

Twelve studies were included to examine the association between “total meat intake” and risk of “advanced prostate cancer” (6, 11, 26, 27, 29, 31–36, 42). Generally, we observed a significant association between “total meat intake” and risk of “advanced prostate cancer” with a summary risk estimate of 1.09 for the highest vs. lowest categories in the fixed-effects model (95% CI: 1.02, 1.16; *I*^2^ = 63.3%, *P* = 0.002) (Figure 4B); however, this relationship was not significant in the random-effects model (RR = 1.05, 95% CI: 0.93, 1.18) (Supplementary Figure 5B). The Egger's test did not show evidence of publication bias (*P* = 0.269). Based on subgroup analyses, we observed that adjustment for alcohol consumption and family history of cancer influenced the association between “total meat intake” and risk of “advanced prostate cancer.” When we did subgroup analysis based on studies that did or did not control for alcohol consumption, we found an increased risk of “advanced prostate cancer” with “total meat consumption” in studies that did adjustment for alcohol intake (RR = 1.24, 95% CI: 1.11, 1.39), while in other studies, there was no significant association (RR = 1.02, 95% CI: 0.95, 1.10). In addition, analysis based on studies that controlled for family history of cancer revealed no significant association in studies that did adjustment for this variable (RR = 1.01, 95% CI: 0.92, 1.11), while others reached a significant positive association between “total meat intake” and risk of “advanced prostate cancer” (RR = 1.17, 95% CI: 1.07, 1.28) (Supplementary Table 2).

### Meta-Analysis on “Total Meat Intake” and “All Outcomes of Prostate Cancer”

**Figure 5:**
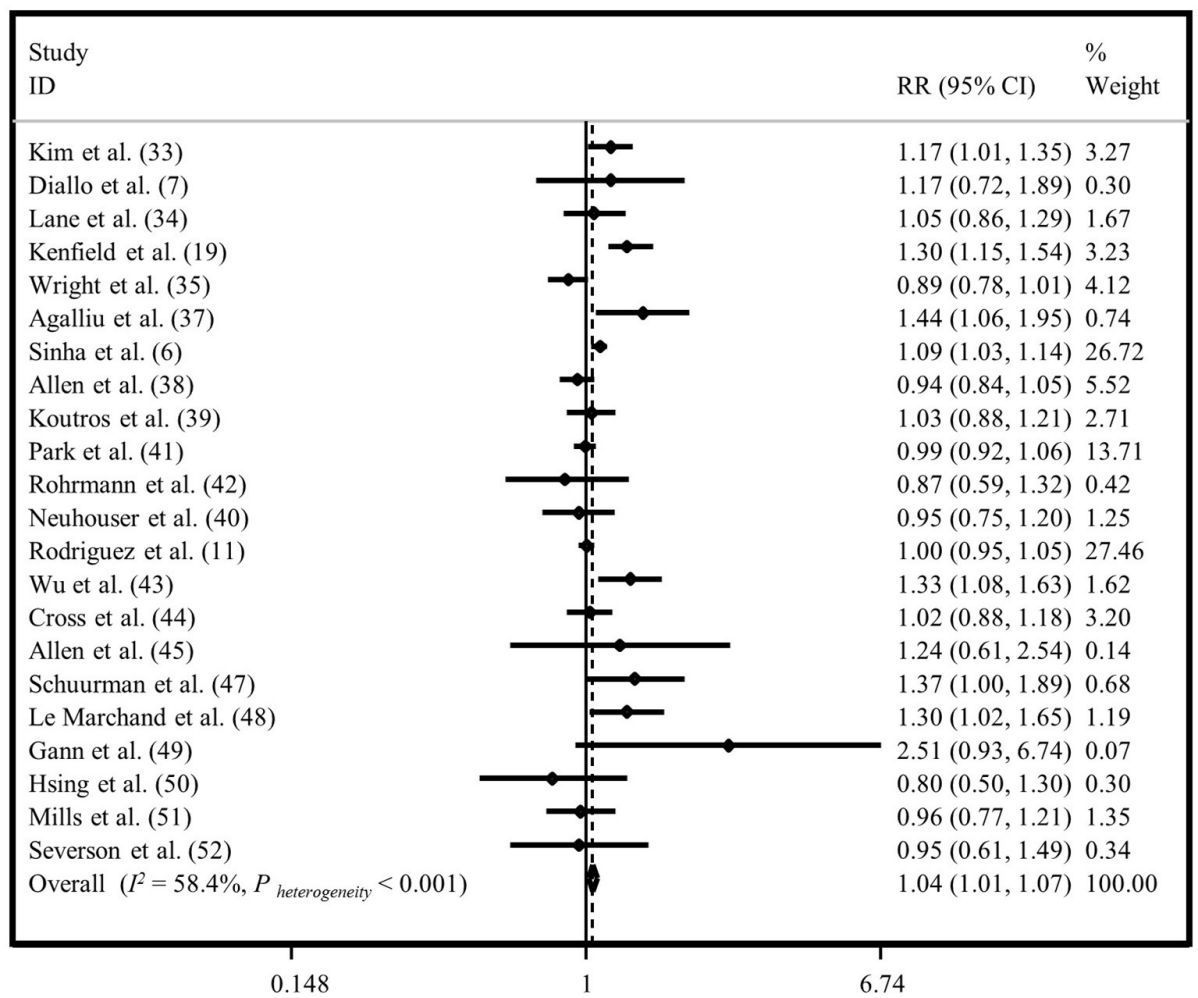
Forest plot derived from fixed-effects meta-analysis investigating the association between total meat intake and all outcomes of prostate cancer. RR, relative risk; CI, confidence intervals; I^2^, I-square.

Twenty-two studies had investigated the association between “total meat intake” and “all outcomes of prostate cancer” (6, 7, 11, 24–27, 29–37, 39–44). The summary risk estimate for “all outcomes of prostate cancer” risk, comparing the highest and lowest “total meat intake,” was 1.04 (95% CI: 1.01, 1.07; *I*^2^ = 58.4%, *P* < 0.001) in the fixed-effects (Figure 5), and 1.06 (95% CI: 1.01, 1.12) in the random-effects analyses (Supplementary Figure 6), indicating that increased intake of “total meat” may be positively associated with an increased risk of “all outcomes of prostate cancer.” Publication bias was not documented by Egger's test (*P* = 0.240).

Subgroup analyses were done to investigate possible sources of heterogeneity. We observed that adjustment for energy intake and alcohol consumption, and study quality score were the possible sources of heterogeneity (Supplementary Table 2).

### Sensitivity Analysis

Findings from sensitivity analyses in each of the above-mentioned meta-analyses revealed that none of the single studies had a significant effect on the pooled effect size.

### Linear and Non-linear Dose-Response Analyses

**Figure 6:**
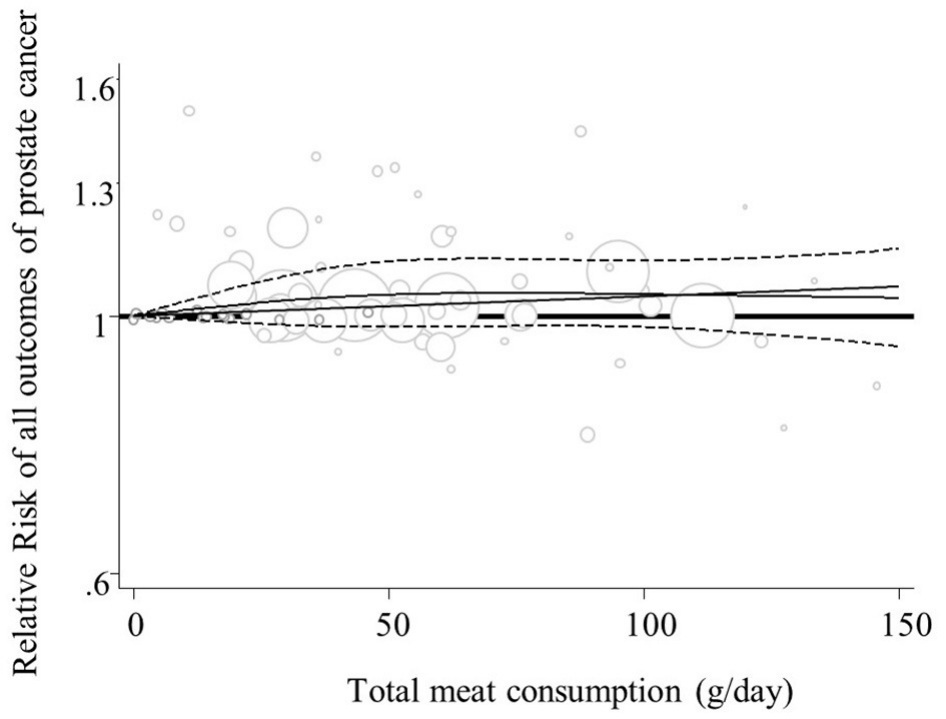
Non-linear dose-response relation between total meat intake and all outcomes of prostate cancer (P-nonlinearity = 0.37; *n* = 18 studies).

Overall, four studies from the linear (27, 35, 40, 41) and seven studies from the non-linear dose-response analysis (23, 26, 27, 35, 38, 40, 41) were excluded and finally, 21 publications in the linear (6, 7, 11, 17, 23–26, 28–34, 37–39, 42–44), and 18 studies in the non-linear analysis (6, 7, 11, 17, 24, 25, 28–34, 37, 39, 42–44) were included. Findings from the linear dose-response analysis for “processed meat intake” and “total prostate cancer” based on twelve prospective studies revealed that consumption of additional 50 grams per day of processed meat might be associated with a 4% increased risk of “total prostate cancer” (RR = 1.04, 95% CI: 1.00, 1.08; *I*^2^ = 0.0%, *P* = 0.51) (Supplementary Figure 8A). No other significant associations were seen between different exposures and study outcomes, either in linear or in non-linear analyses (Figure 6; Supplementary Figures 7, 8B, 9–15).

## Discussion

In this systematic review and meta-analysis of prospective studies, we found that total meat intake was marginally associated with all outcomes of prostate cancer risk. This association was more evident about processed meat consumption. Although a significant weak relationship was observed between red meat consumption and risk of total prostate cancer in the fixed-effects model, there was no such significant association between red meat consumption and risk of advanced prostate cancer.

There were an estimated 1.3 million new cases of prostate cancer and 3,59,000 deaths worldwide in 2018 (1). In the present systematic review and meta-analysis, we found that dietary intake of processed meat might be associated with a greater risk of total prostate cancer. Although such a significant positive relationship was observed with advanced prostate cancer in the fixed-effects analysis, it was not significant in the random-effects analysis. Moreover, we observed a weak significant linear dose-response association between processed meat intake and total prostate cancer. Three earlier studies have previously investigated the association between processed meat consumption and risk of prostate cancer (12–14). In line with our study, a marginally significant dose-response association between processed meat intake and total prostate cancer was reported by Alexander et al. (12). Also Bylsma and Alexander have reported a significant positive relationship between consumption of processed meat and risk of total prostate cancer in their meta-analysis (13). However, no significant relationship was observed between processed meat intake and risk of advanced prostate cancer in that meta-analysis (13). In addition, in a pooled analysis of 15 prospective cohort studies (14) and a meta-analysis of Alexander et al., no significant relationship was found between processed meat consumption and risk of prostate cancer (12). Different findings might be explained by some reasons. For example, in the present study, we included three additional studies that were not included in previous publications (7, 25, 26). Furthermore, prior studies had not combined data on fatal prostate cancer and advanced prostate cancer, while we considered all as advanced prostate cancer.

Although we did not observe a significant association between red meat intake and risk of advanced prostate cancer, a significant weak relationship between red meat consumption and risk of total prostate cancer was seen in the fixed-effects analysis. Previous studies reported no significant association between red meat intake and risk of total and advanced prostate cancer (12–14). Discrepant findings might be originated from the inclusion of two new studies in our analysis (7, 25). In addition, we excluded the study of Veierød et al., which was done on men aged under 18 years (22), while it was included in the previous meta-analysis. Moreover, we included the study of Neuhouser et al. (32), in which type of meat was not specified, in the category of “total meat” analysis, while earlier studies had considered this study in their analysis on “total red meat intake.”

When we combined red and processed meat intake, no significant association was found with total and advanced prostate cancer. This might be attributed to the inclusion of studies that examined both red meat and processed meat intakes, and lack of inclusion of studies that examined only red meat or processed meat consumption.

The present meta-analysis has several strengths overs previous meta-analyses. This study pooled effect sizes from 22 papers to investigate the link between total meat intake and risk of all outcomes of prostate cancer for the first time. In addition, we included five new big cohort studies in this meta-analysis. Furthermore, additional studies were included in the linear and non-linear dose-response analyses in the current study. Moreover, both random-effects and fixed-effects models were done in this study to investigate more accurate association between meat intake and prostate cancer. Also, further subgroup analyses, as compared with earlier studies, was performed in this analysis. Finally, in additional to linear dose-response analysis, we did non-linear dose-response analysis in this study as well, while previous studies have only performed linear dose-response analysis.

The relationship between meat consumption and risk of prostate cancer can be explained by several potential mechanisms. Heme iron in red and processed meat (54) and N-nitroso compounds (NOCs) in processed meat are considered as DNA damaging factors (55). Heme iron, which is carried by hemoglobin or directly via the bloodstream throughout the body, is able to catalyze the oxidative reactions that might cause DNA, protein, and lipid oxidations in multiple organs including prostate (55). NOCs in processed meat are formed by the reaction between nitrites or nitrates and amines or amides (56), and the presence of NOCs in processed meat may increase the risk of cancers (8). The presence of heterocyclic amines (HCAs) and polycyclic aromatic hydrocarbons (PAHs) in cooked foods, particularly meat, and their excessive consumption may increase the risk of some types of cancer (57). HCAs are part of a family of mutagenic compounds and are believed to play an important role in the etiology of human cancers. It has been shown that 2-Amino-1-methyl-6-phenylimiazo[4,5-b]pyridine (PhIP), but not other prominent HCAs existing in cooked meats, forms DNA adducts in the human prostate, which can, in turn, led to abnormal prostate cells (58). Also, excess fat in meat increases the production of hormones such as estrogens, which may further increase the risk of hormone-related cancers such as breast and prostate cancer (59). As shown in previous publications, high dietary intake of red and processed meat was associated with increased risk of depression (60), which can in turn elevate the risk of prostate cancer (61).

This study has several strengths. For the first time, we performed a non-linear dose-response association between meat intake and risk of prostate cancer. We included only prospective studies in this meta-analysis. Therefore, the probability of recall and selection bias is minimized, however, one should consider lost to follow-up in each individual study. In the current analysis a few studies have reported number of people lost to follow-up. This should be considered in the interpretation of our findings. In the sensitivity analysis, our findings were stable and robust. Most included studies had controlled for confounders such as age, energy intake, and smoking. We had some limitations in this meta-analysis as well. In most studies, FFQ has been used to assess food intake, therefore, measurement error and misclassification of study subjects was possible. We did not examine the association between cooking methods of meat and prostate cancer. In some studies, processed poultry was also included in total processed meat.

In conclusion, we found that total meat intake might be poorly associated with all outcomes of prostate cancer. Consumption of processed meat might be associated with an increased risk of total and advanced prostate cancer. Also, we observed a weak relationship between red meat consumption and risk of total prostate cancer, but not with advanced prostate cancer. Given some significant, albeit weak, associations between meat consumption and risk of different types of prostate cancer, recommendations on the consumption of meat should be done cautiously. In addition, consumption of processed meat intake, due to its detrimental effects on human health, should be reduced. Policymakers might use the current findings to make policies about reducing the production and availability of processed meats. In addition, it seems that subsidizing red and processed meats should be shifted toward healthier animal protein options such as white meat and dairy products. In order to increase consumer awareness, the possible risks of consuming processed meat could be mentioned in nutrition labels.

## Data Availability Statement

The original contributions presented in the study are included in the article/Supplementary Materials, further inquiries can be directed to the corresponding author.

## Author Contributions

SN-M, AS-M, and AA contributed to the conception, design, literature search, statistical analyses, data interpretation, and manuscript drafting. BL contributed to the conception, design, and manuscript drafting. AE contributed to the conception, design, statistical analyses, data interpretation, manuscript drafting, and supervised the study. All authors approved the final manuscript for submission.

## Conflict of Interest

The authors declare that the research was conducted in the absence of any commercial or financial relationships that could be construed as a potential conflict of interest.

## Publisher's Note

All claims expressed in this article are solely those of the authors and do not necessarily represent those of their affiliated organizations, or those of the publisher, the editors and the reviewers. Any product that may be evaluated in this article, or claim that may be made by its manufacturer, is not guaranteed or endorsed by the publisher.

## Supplementary Material

Supplementary Figure 1 Forest plot derived from random-effects meta-analysis investigating the association between red meat intake and risk of total prostate cancer **(A)** and advanced prostate cancer **(B)**. RR, relative risk; CI, confidence intervals; I^2^, I-square.

Supplementary Figure 2 Forest plot derived from random-effects meta-analysis investigating the association between processed meat intake and risk of total prostate cancer **(A)** and advanced prostate cancer **(B)**. RR, relative risk; CI, confidence intervals; I^2^, I-square.

Supplementary Figure 3 Forest plot derived from fixed-effects meta-analysis investigating the association between red and processed meat intake and risk of total prostate cancer **(A)** and advanced prostate cancer **(B)**. RR, relative risk; CI, confidence intervals; I^2^, I-square.

Supplementary Figure 4 Forest plot derived from random-effects meta-analysis investigating the association between red and processed meat intake and risk of total prostate cancer **(A)** and advanced prostate cancer **(B)**. RR, relative risk; CI, confidence intervals; I^2^, I-square.

Supplementary Figure 5 Forest plot derived from random-effects meta-analysis investigating the association between total meat intake and risk of total prostate cancer **(A)** and advanced prostate cancer **(B)**. RR, relative risk; CI, confidence intervals; I^2^, I-square.

Supplementary Figure 6 Forest plot derived from random-effects meta-analysis investigating the association between total meat intake and all outcomes of prostate cancer. RR, relative risk; CI, confidence intervals; I^2^, I-square.

Supplementary Figure 7 Summary of relative risk of total prostate cancer **(A)** and advanced prostate cancer **(B)** for each 50 g/day increase in red meat intake. CI, confidence intervals; I^2^, I-square.

Supplementary Figure 8 Summary of relative risk of total prostate cancer **(A)** and advanced prostate cancer **(B)** for each 50 g/day increase in processed meat intake. CI, confidence intervals; I^2^, I-square.

Supplementary Figure 9 Summary of relative risk of total prostate cancer **(A)** and advanced prostate cancer **(B)** for each 50 g/day increase in red and processed meat intake. CI, confidence intervals; I^2^, I-square.

Supplementary Figure 10 Summary of relative risk of total prostate cancer **(A)** and advanced prostate cancer **(B)** for each 50 g/day increase in total meat intake. CI, confidence intervals; I^2^, I-square.

Supplementary Figure 11 Summary of relative risk of all outcomes of prostate cancer for each 50 g/day increase in total meat intake. CI, confidence intervals; I^2^, I-square.

Supplementary Figure 12 Non-linear dose-response relation between red meat intake and total prostate cancer **(A)** (P-non-linearity = 0.76; *n* = 11 studies) and advanced prostate cancer **(B)** (P-non-linearity = 0.40; *n* = 8 studies).

Supplementary Figure 13 Non-linear dose-response relation between processed meat intake and total prostate cancer **(A)** (P-non-linearity = 0.13; *n* = 11 studies) and advanced prostate cancer **(B)** (P-nonlinearity = 0.81; n = 7 studies).

Supplementary Figure 14 Nonlinear dose-response relation between red and processed meat intake and total prostate cancer **(A)** (P-non-linearity = 0.35; *n* = 10 studies) and advanced prostate cancer **(B)** (P-non-linearity = 0.86; *n* = 8 studies).

Supplementary Figure 15 Non-linear dose-response relation between total meat intake and total prostate cancer **(A)** (P-nonlinearity = 0.41; *n* = 16 studies) and advanced prostate cancer **(B)** (P-non-linearity = 0.26; *n* = 10 studies).

Supplementary Table 1 Characteristics of included studies in the systematic review on meat consumption and risk of prostate cancer.

Supplementary Table 2 Results of subgroup analyses on the association of meat consumption and risk of prostate cancer.
