# Supplementary figures and images for: Association Between Red and Processed Meat Consumption and Risk of Prostate Cancer: A Systematic Review and Meta-Analysis
Source: Front Nutr. 2022 Feb 7;9:801722. doi: 10.3389/fnut.2022.801722 (PMC8859108; doi:10.3389/fnut.2022.801722)

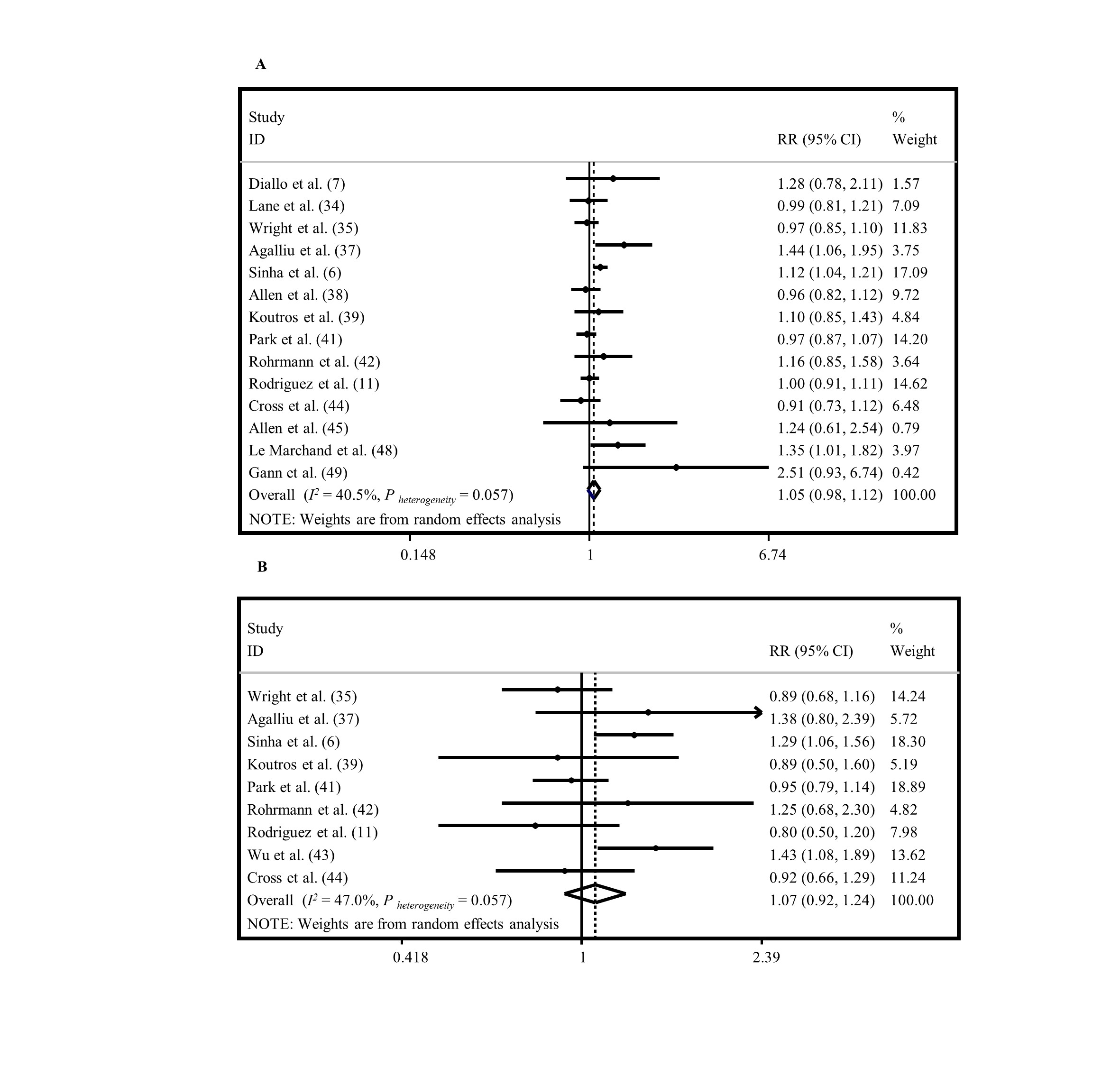

Supplement: Supplementary Figure 1 — Forest plot derived from random-effects meta-analysis investigating the association between red meat intake and risk of total prostate cancer (A) and advanced prostate cancer (B). RR, relative risk; CI, confidence intervals; I2, I-square. [file Image_1.jpg]

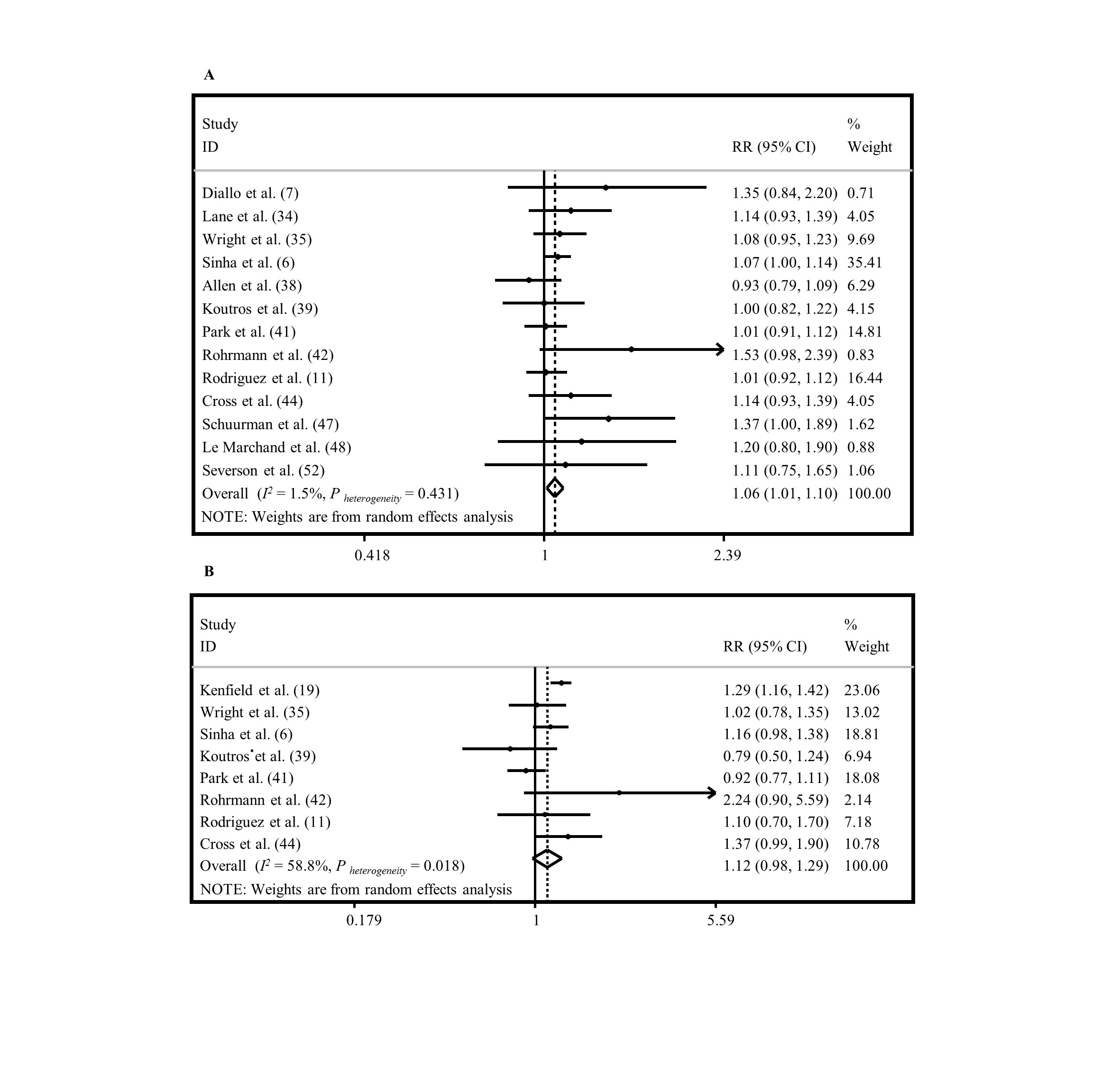

Supplement: Supplementary Figure 2 — Forest plot derived from random-effects meta-analysis investigating the association between processed meat intake and risk of total prostate cancer (A) and advanced prostate cancer (B). RR, relative risk; CI, confidence intervals; I2, I-square. [file Image_2.jpg]

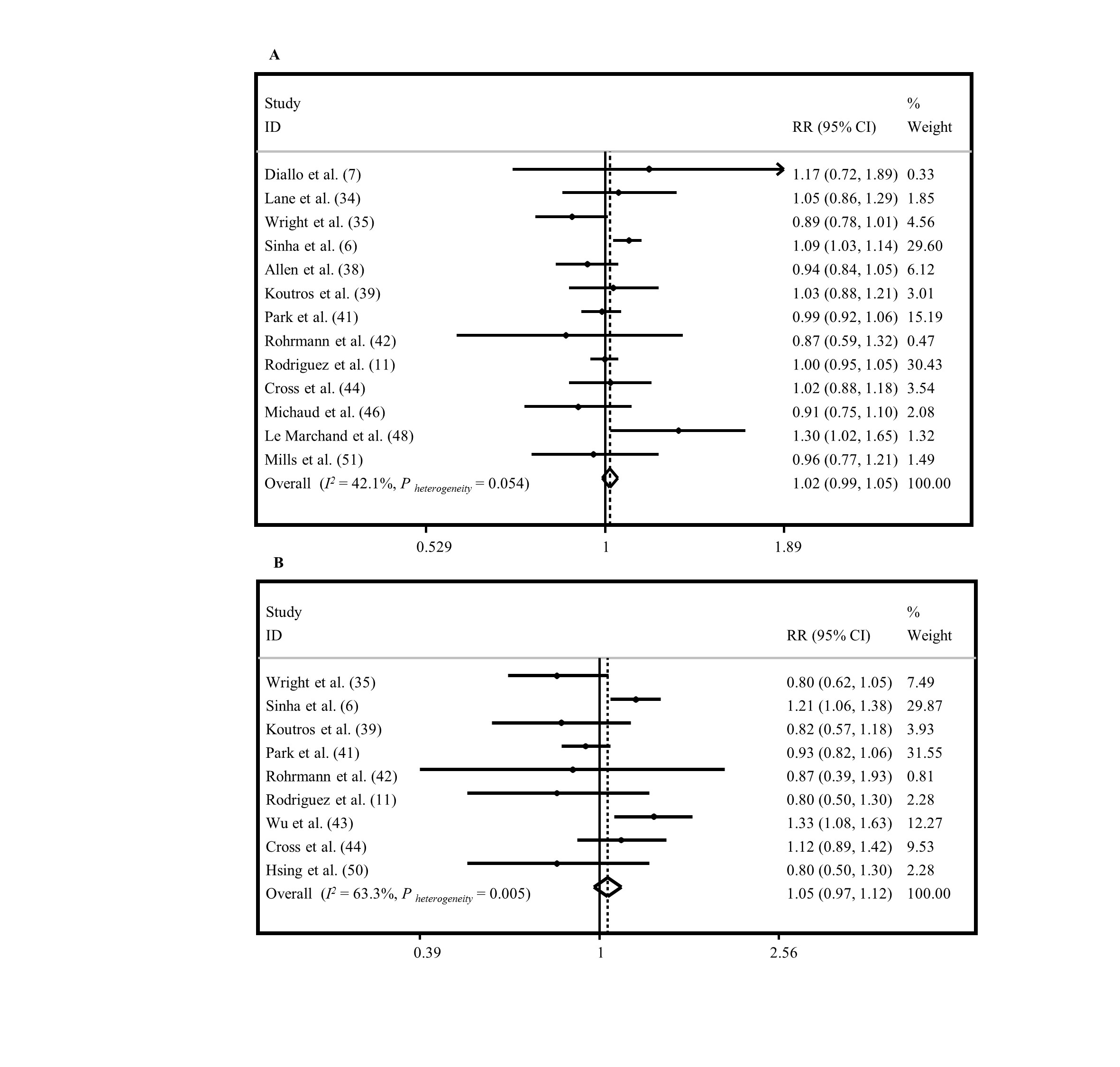

Supplement: Supplementary Figure 3 — Forest plot derived from fixed-effects meta-analysis investigating the association between red and processed meat intake and risk of total prostate cancer (A) and advanced prostate cancer (B). RR, relative risk; CI, confidence intervals; I2, I-square. [file Image_3.jpg]

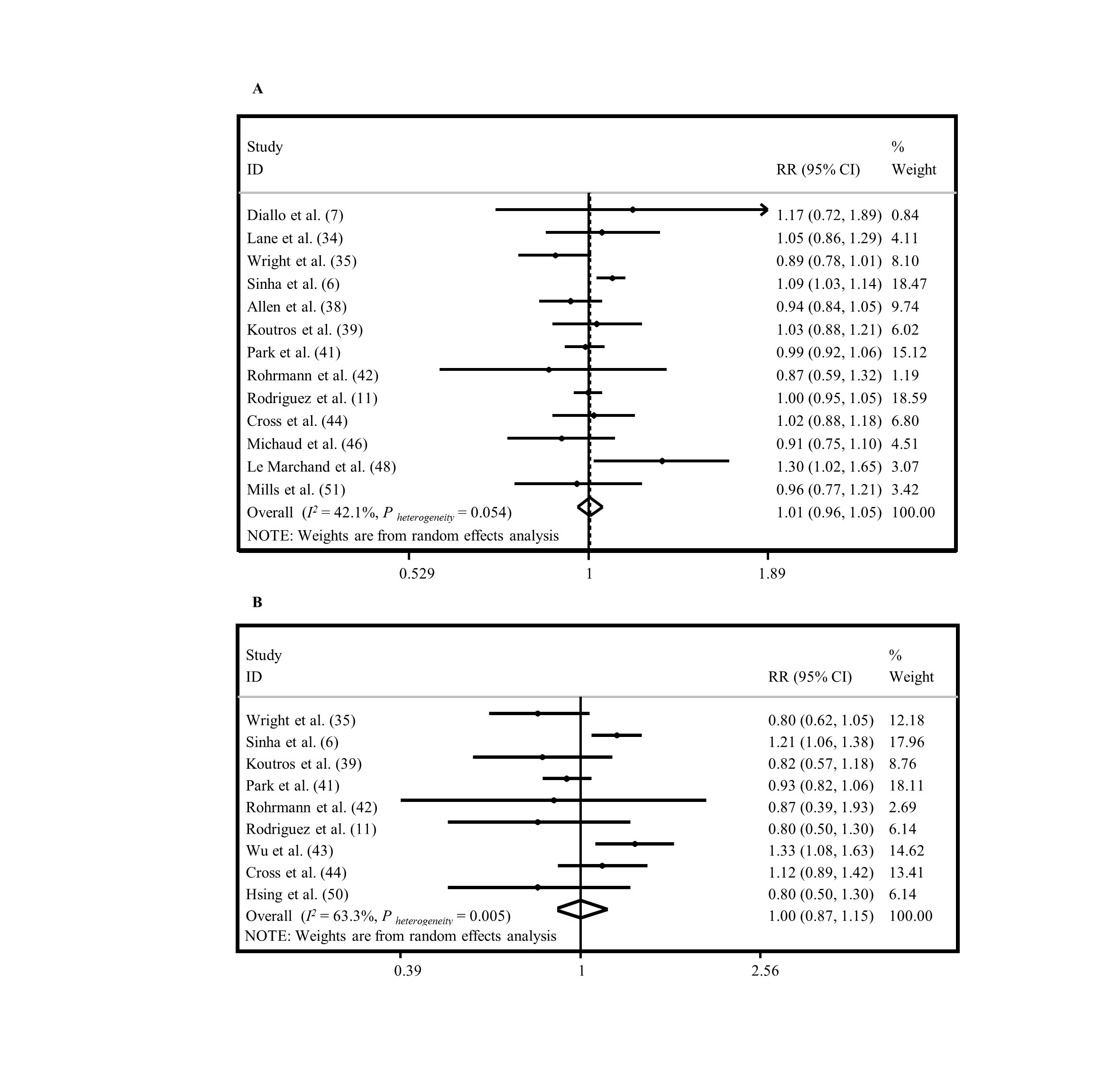

Supplement: Supplementary Figure 4 — Forest plot derived from random-effects meta-analysis investigating the association between red and processed meat intake and risk of total prostate cancer (A) and advanced prostate cancer (B). RR, relative risk; CI, confidence intervals; I2, I-square. [file Image_4.jpg]

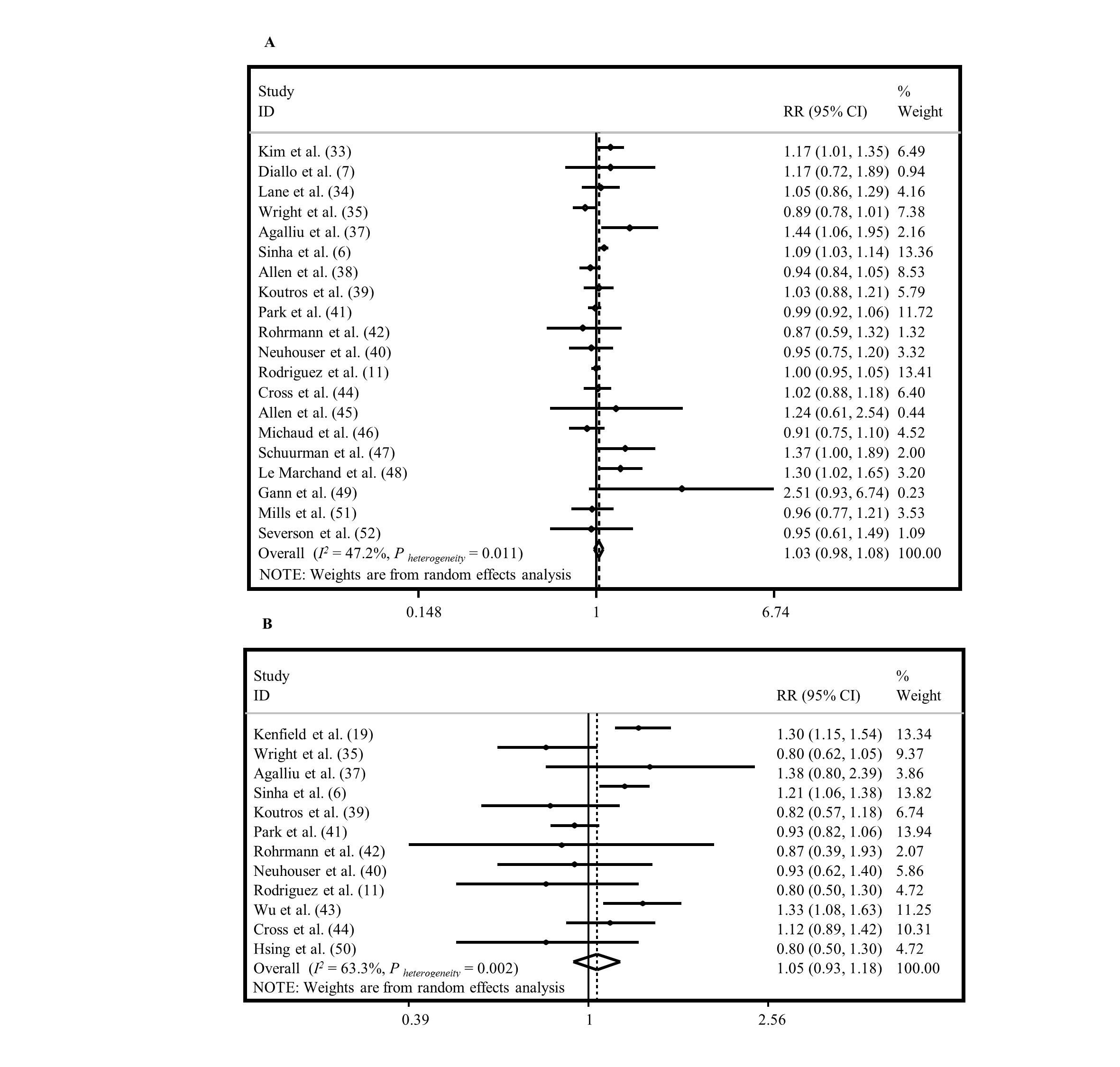

Supplement: Supplementary Figure 5 — Forest plot derived from random-effects meta-analysis investigating the association between total meat intake and risk of total prostate cancer (A) and advanced prostate cancer (B). RR, relative risk; CI, confidence intervals; I2, I-square. [file Image_5.jpg]

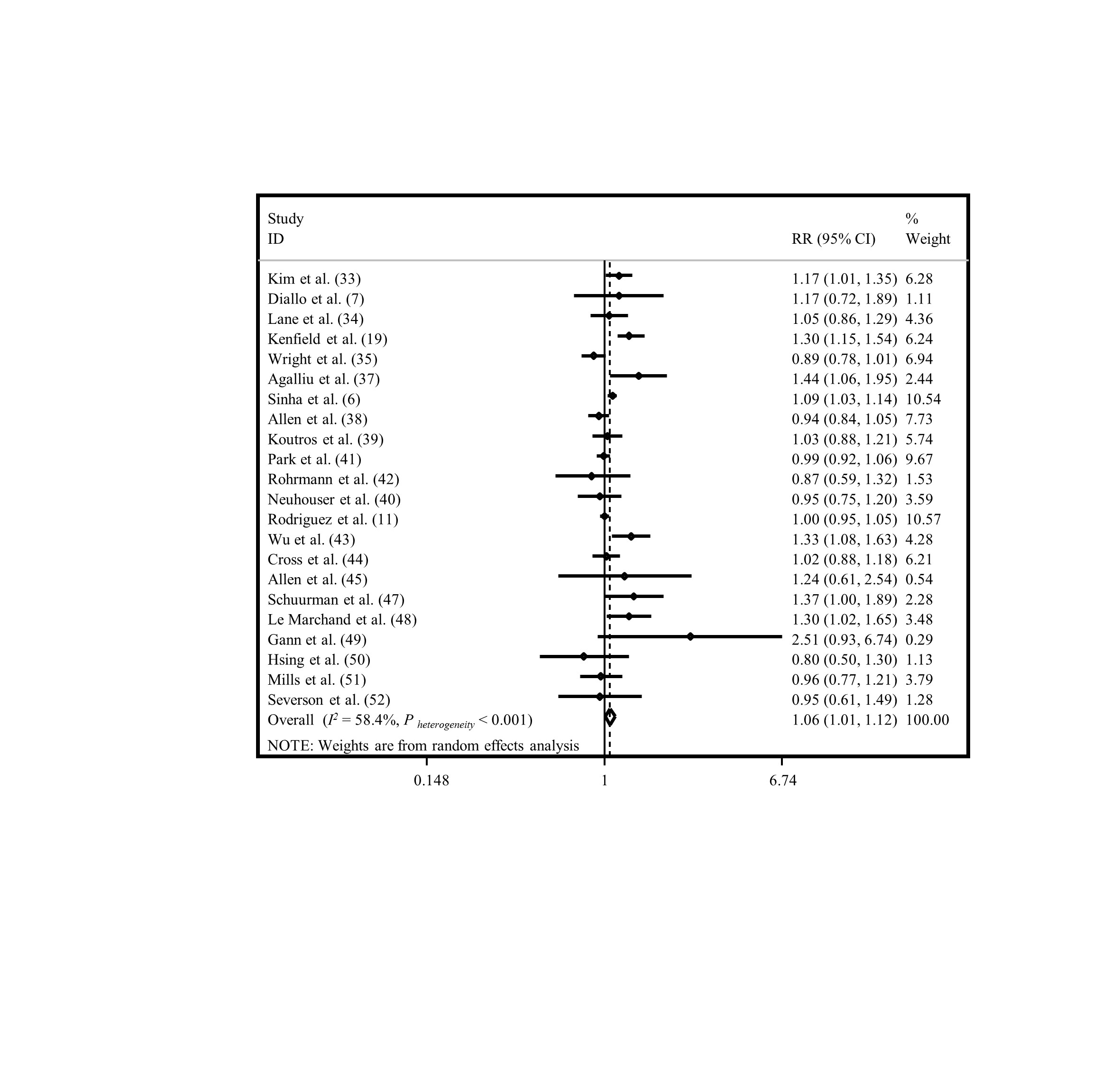

Supplement: Supplementary Figure 6 — Forest plot derived from random-effects meta-analysis investigating the association between total meat intake and all outcomes of prostate cancer. RR, relative risk; CI, confidence intervals; I2, I-square. [file Image_6.jpg]

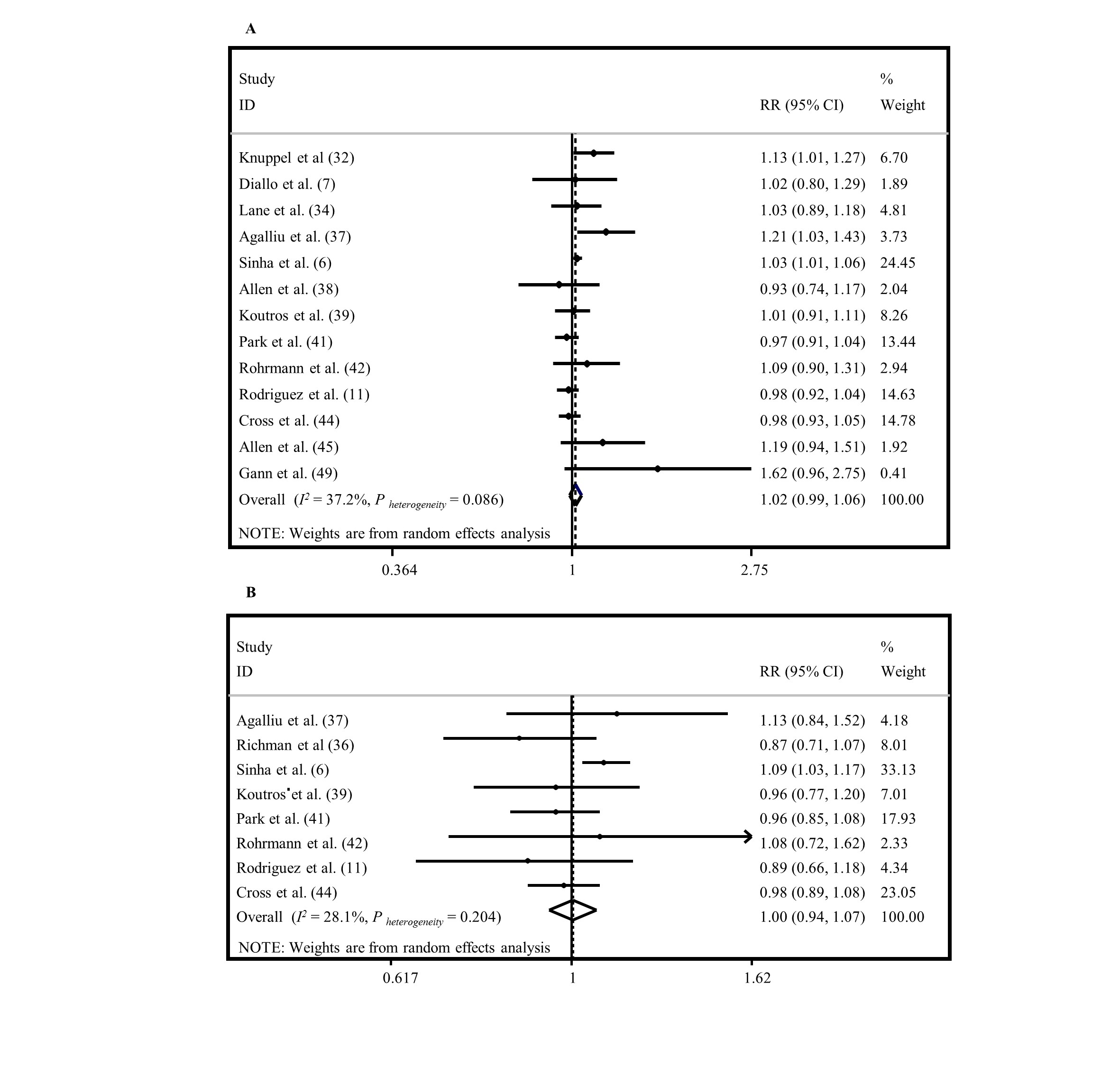

Supplement: Supplementary Figure 7 — Summary of relative risk of total prostate cancer (A) and advanced prostate cancer (B) for each 50 g/day increase in red meat intake. CI, confidence intervals; I2, I-square. [file Image_7.jpg]

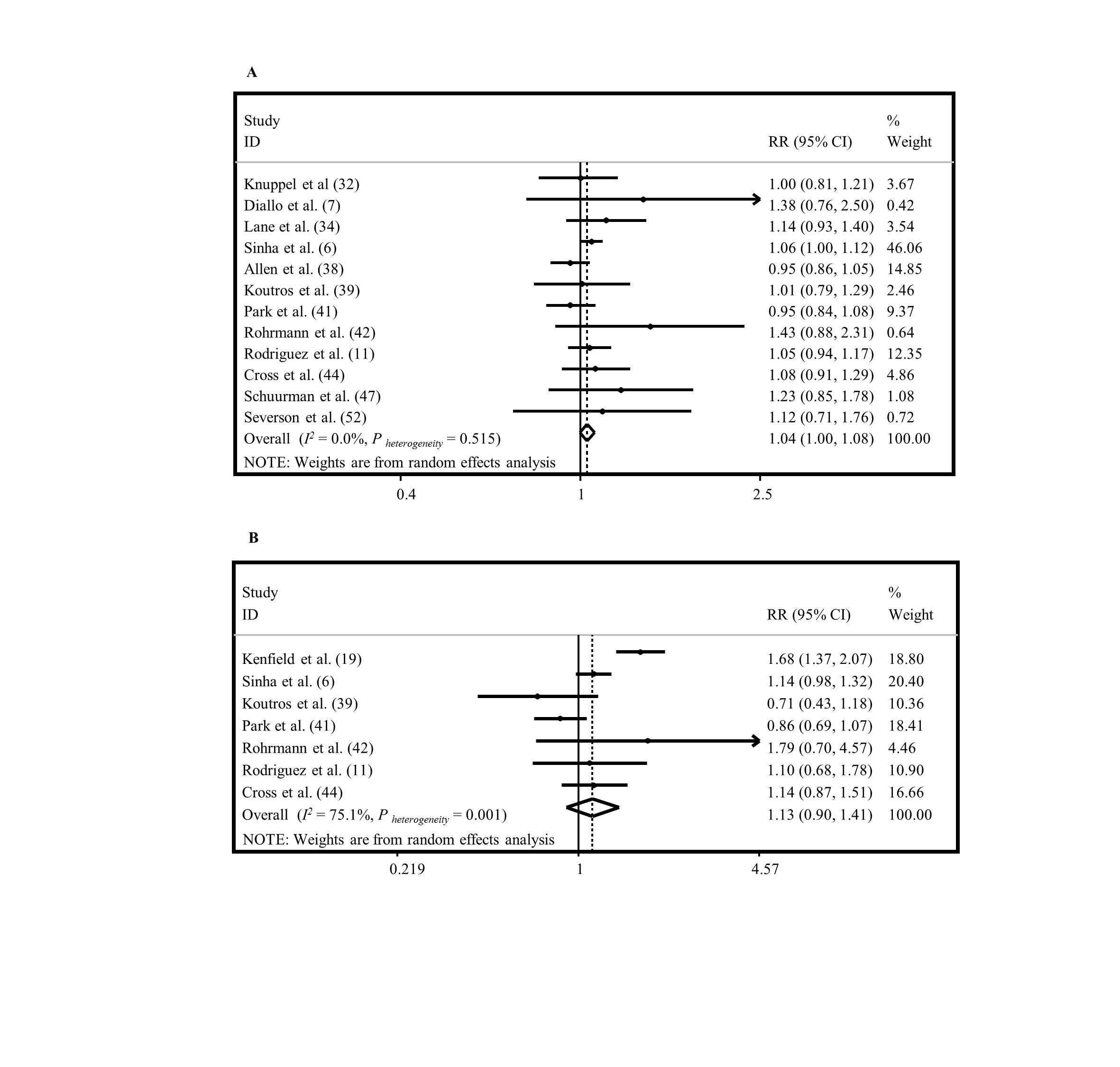

Supplement: Supplementary Figure 8 — Summary of relative risk of total prostate cancer (A) and advanced prostate cancer (B) for each 50 g/day increase in processed meat intake. CI, confidence intervals; I2, I-square. [file Image_8.jpg]

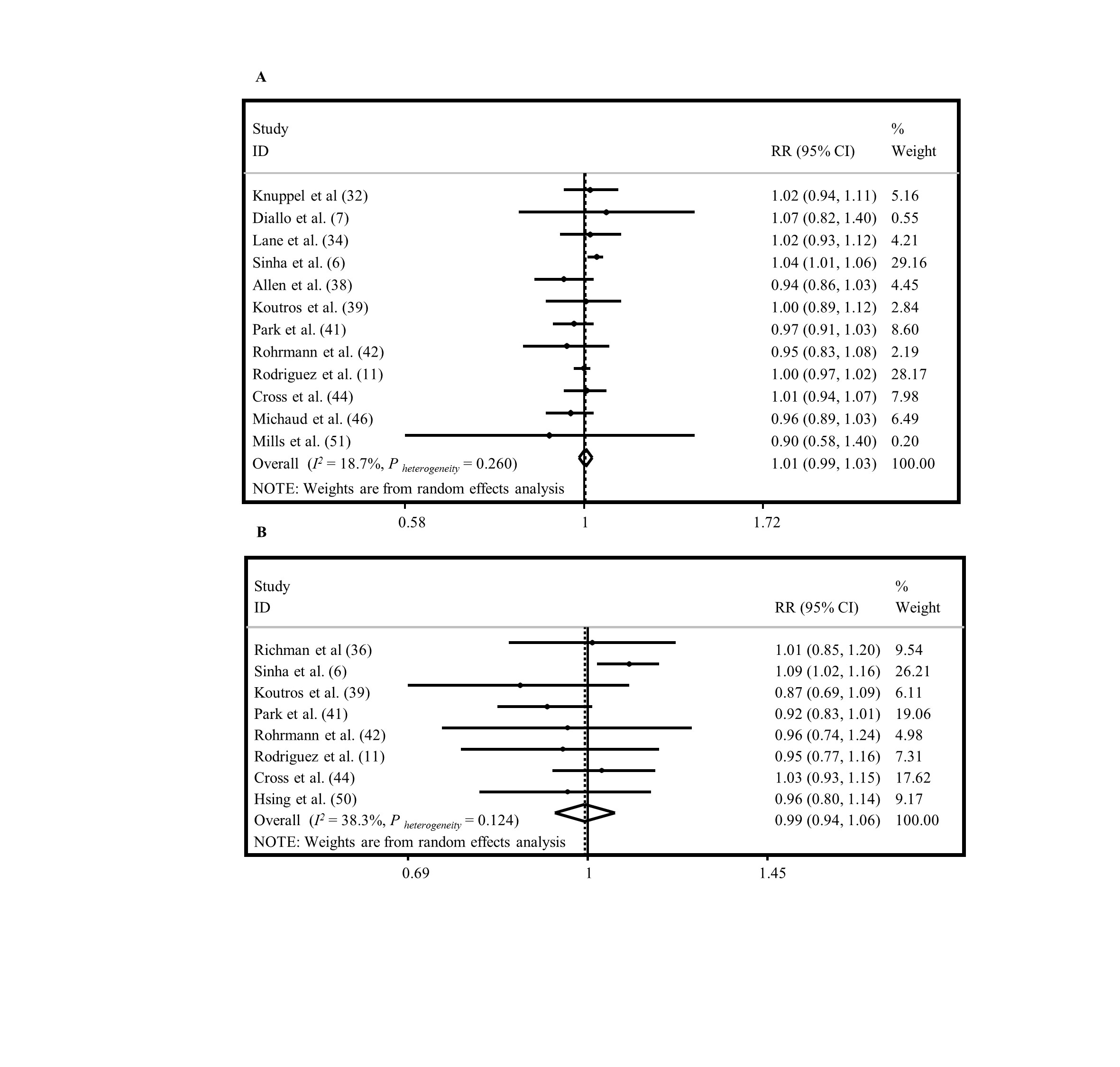

Supplement: Supplementary Figure 9 — Summary of relative risk of total prostate cancer (A) and advanced prostate cancer (B) for each 50 g/day increase in red and processed meat intake. CI, confidence intervals; I2, I-square. [file Image_9.jpg]

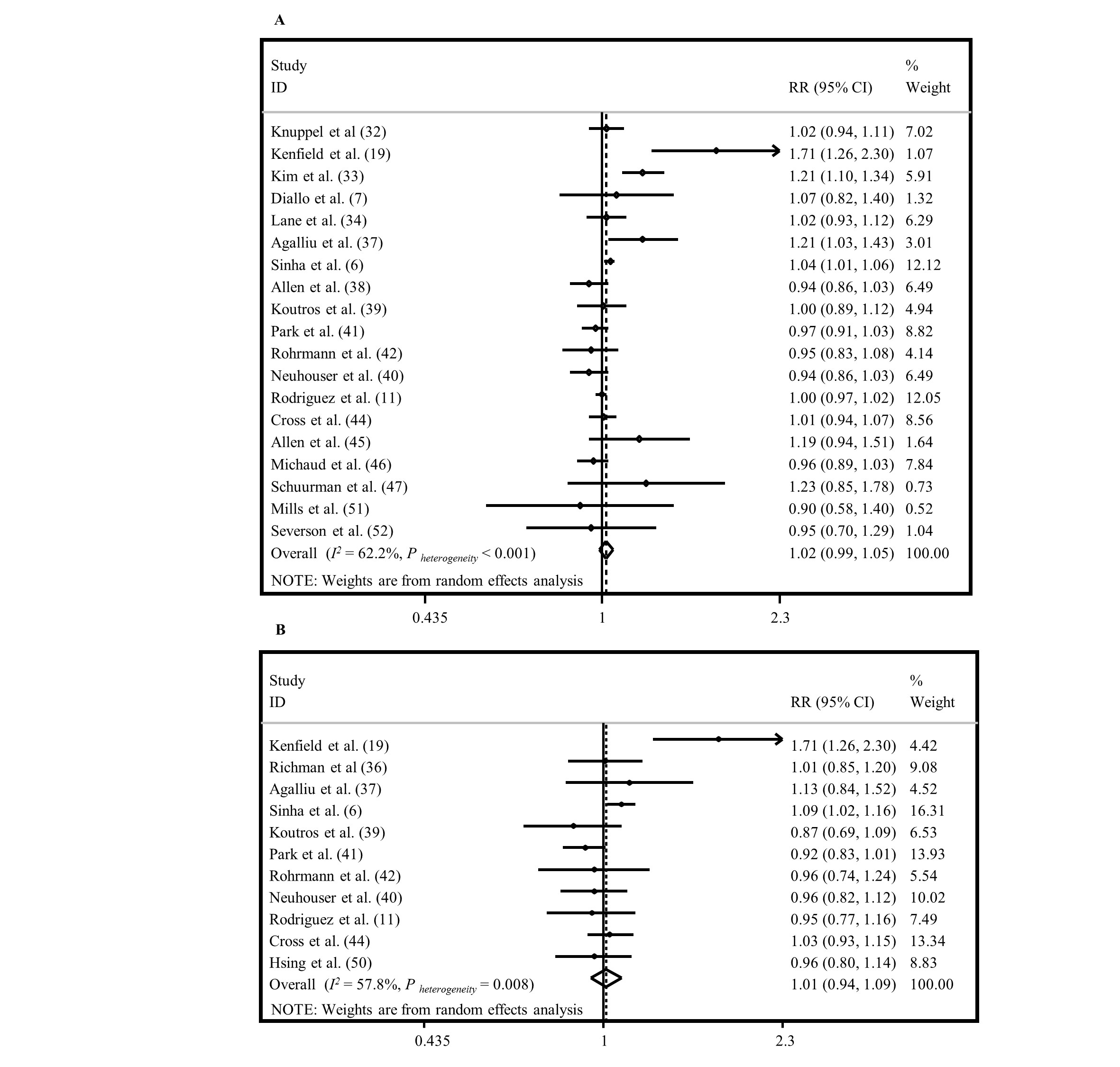

Supplement: Supplementary Figure 10 — Summary of relative risk of total prostate cancer (A) and advanced prostate cancer (B) for each 50 g/day increase in total meat intake. CI, confidence intervals; I2, I-square. [file Image_10.jpg]

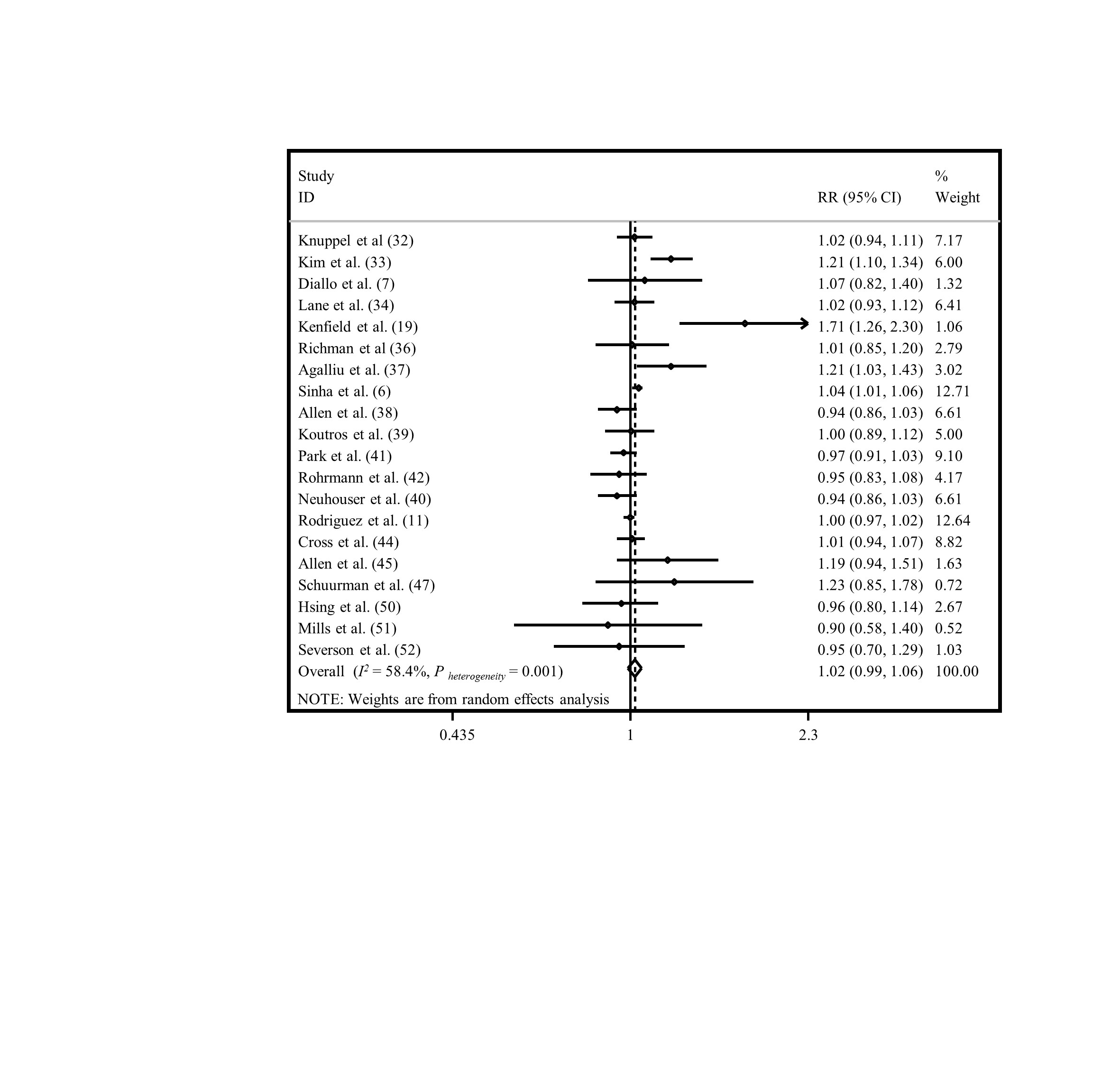

Supplement: Supplementary Figure 11 — Summary of relative risk of all outcomes of prostate cancer for each 50 g/day increase in total meat intake. CI, confidence intervals; I2, I-square. [file Image_11.jpg]

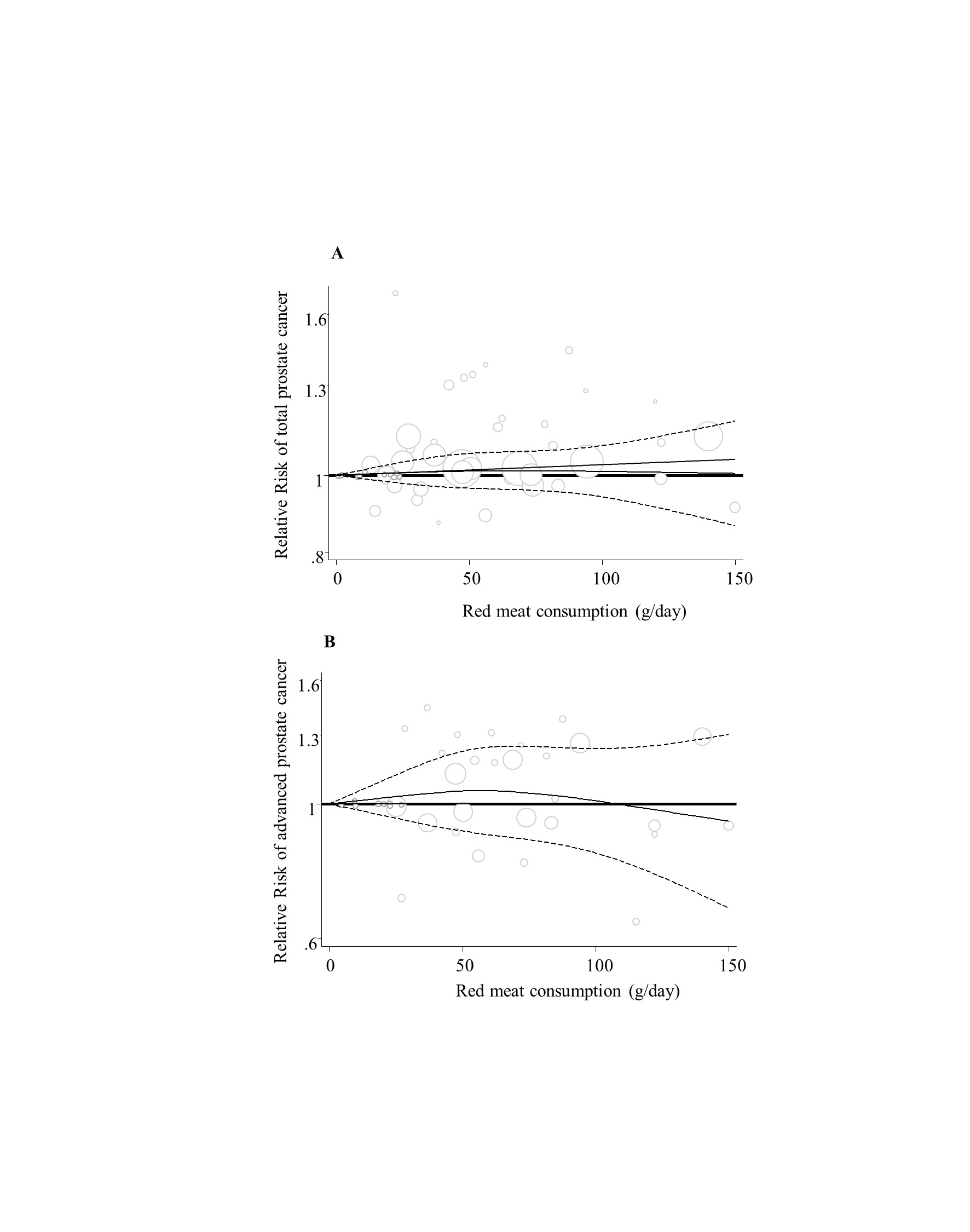

Supplement: Supplementary Figure 12 — Non-linear dose-response relation between red meat intake and total prostate cancer (A) (P-non-linearity = 0.76; n = 11 studies) and advanced prostate cancer (B) (P-non-linearity = 0.40; n = 8 studies). [file Image_12.jpg]

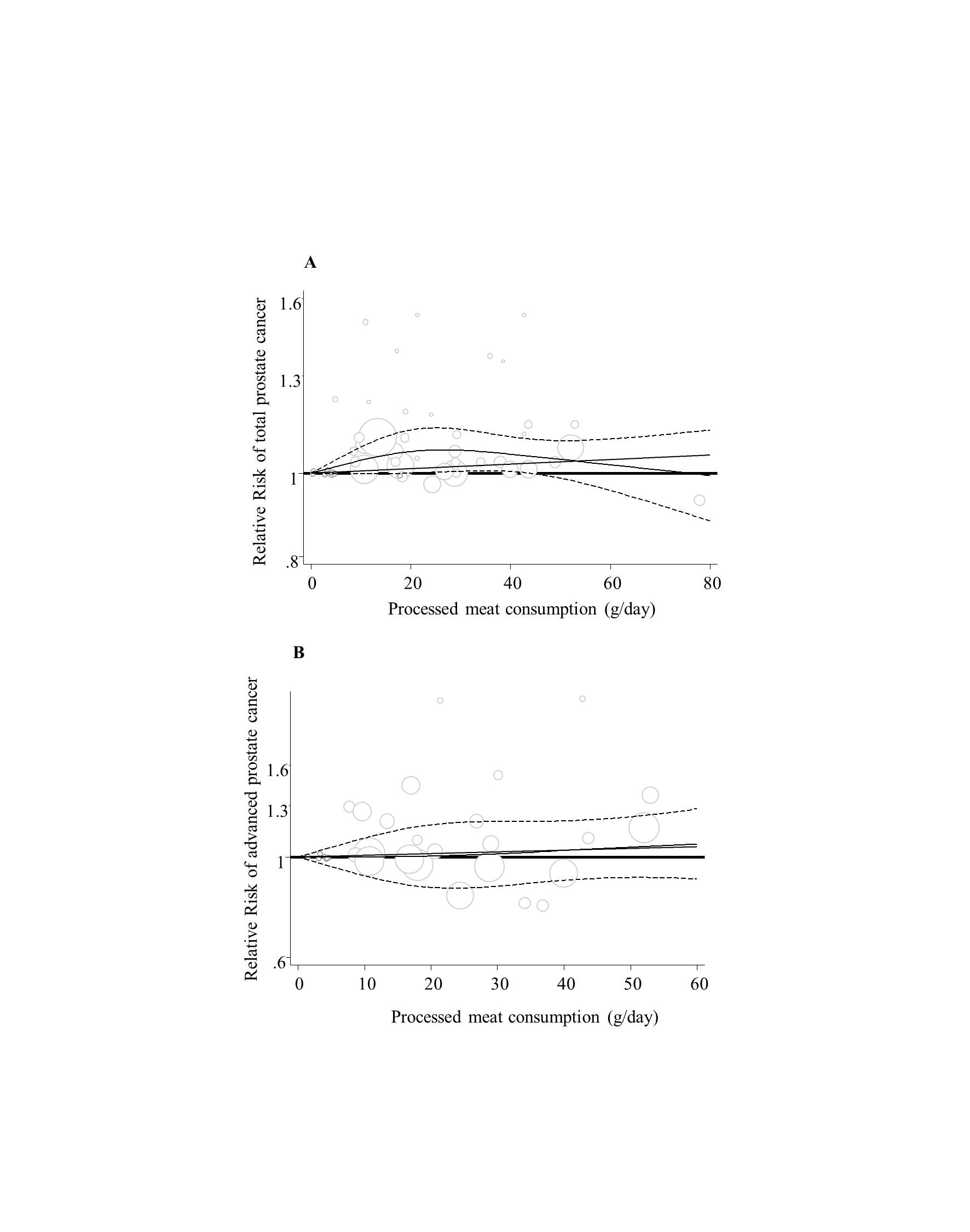

Supplement: Supplementary Figure 13 — Non-linear dose-response relation between processed meat intake and total prostate cancer (A) (P-non-linearity = 0.13; n = 11 studies) and advanced prostate cancer (B) (P-nonlinearity = 0.81; n = 7 studies). [file Image_13.jpg]

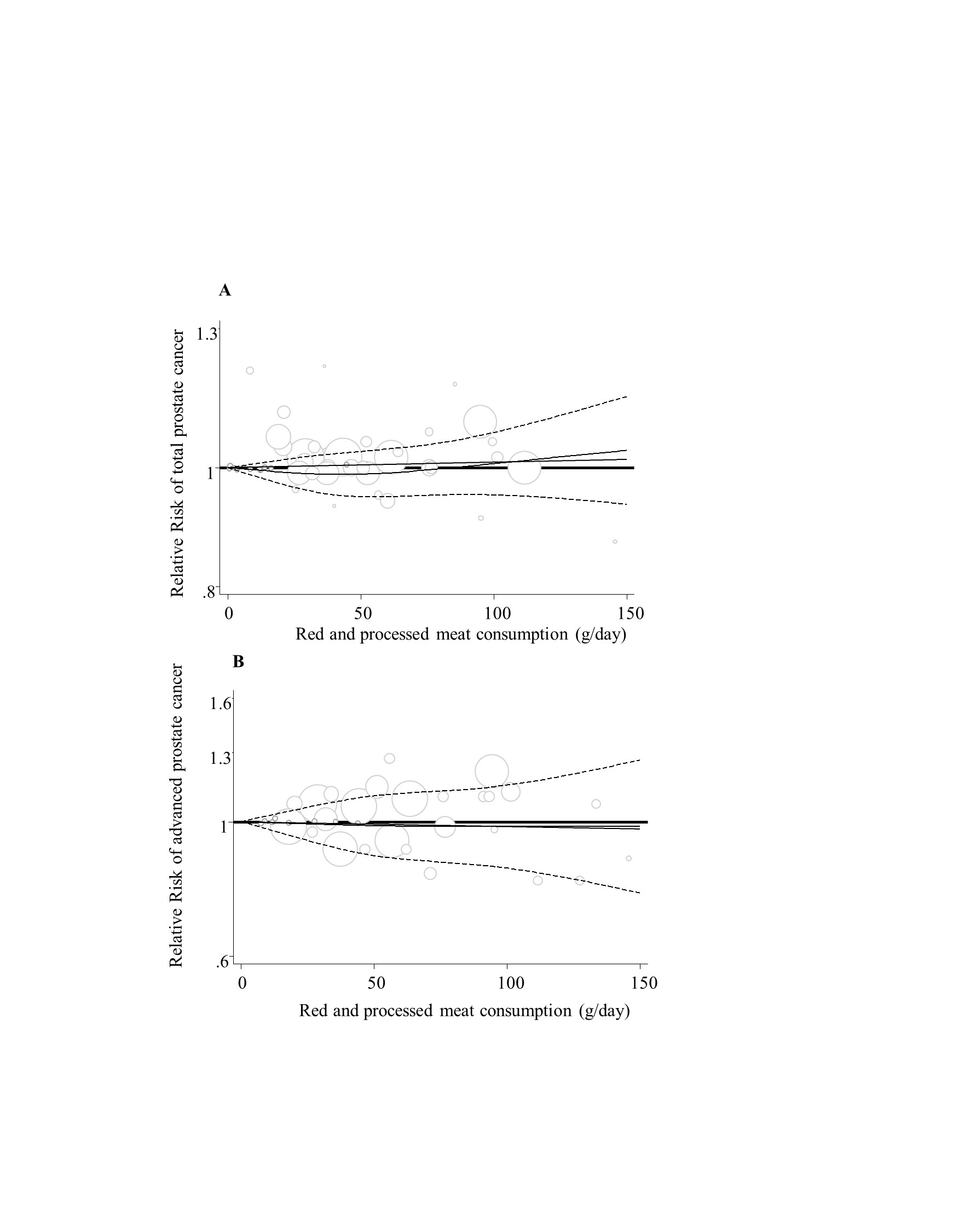

Supplement: Supplementary Figure 14 — Nonlinear dose-response relation between red and processed meat intake and total prostate cancer (A) (P-non-linearity = 0.35; n = 10 studies) and advanced prostate cancer (B) (P-non-linearity = 0.86; n = 8 studies). [file Image_14.jpg]

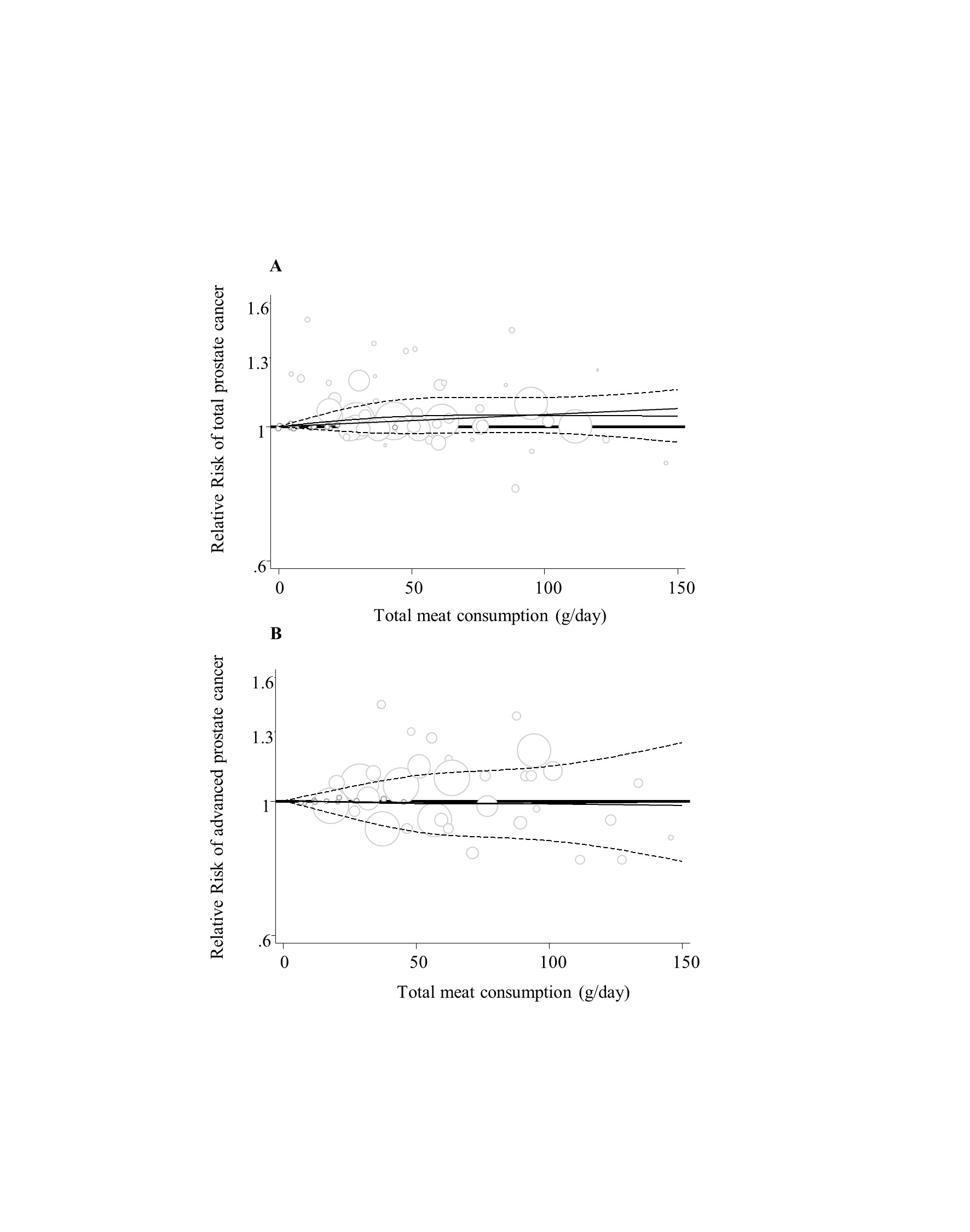

Supplement: Supplementary Figure 15 — Non-linear dose-response relation between total meat intake and total prostate cancer (A) (P-nonlinearity = 0.41; n = 16 studies) and advanced prostate cancer (B) (P-non-linearity = 0.26; n = 10 studies). [file Image_15.jpg]
