# Supplementary material for: Association Between Red and Processed Meat Consumption and Risk of Prostate Cancer: A Systematic Review and Meta-Analysis
Source: Front Nutr. 2022 Feb 7;9:801722. doi: 10.3389/fnut.2022.801722 (PMC8859108; doi:10.3389/fnut.2022.801722)
Supplement: Supplementary Table 1 — Characteristics of included studies in the systematic review on meat consumption and risk of prostate cancer. [file Table_1.DOCX]

| **Supplementary Table 1. Characteristics of included studies in the systematic review on meat consumption and risk of prostate cancer** | | | | | | | | | | |
| --- | --- | --- | --- | --- | --- | --- | --- | --- | --- | --- |
| **First author, (reference)** | **Cohort name/country** | **Mean age or age range, years** | ***No*. of subjects (*no*. of cases)** | **Follow up, years** | **Assessment of exposure** | **Exposure** | **Outcome** | **Comparison** | **RR**  **(95% CI)** | **Adjustments** |
| **Red meat consumption** | | | | | | | | | | |
| **Total prostate cancer** | | | | | | | | | | |
| Knuppel et al. (32) | National Health Service (NHS)/ England | 37-73 | 219,039 (5,958) | 6.9 | 24 h-dietary recall | Red meat | Prostate cancer | Per 50 g/day | 1.13 ( 1.01, 1.27) | 1, 2, 4, 11, 13, 17, 19, 55, 56, 45, 60, 61, 62, 63, 64, 65, 66, 67 |
| Diallo et al.  (7) | NutriNet-Santé study/France | ≥35 | 15,546  (222) | 7 | 24 h-dietary records | Red meat | Prostate cancer | 93.9±42 vs. 3.6±6.7 g/d | 1.28 (0.78, 2.11) | 1, 2, 4, 7, 8, 9, 11, 13, 17, 20, 40, 50, 55, 56, 57 |
| Lane et al.  (34) | EPIC-Norfolk, EPIC-Oxford, NSHD, Whitehall II, and ProtecT/UK | Cases:  63  Controls:  62.7 | Cases:  1,717  Controls:  3,528 | 6.6-13.3 | Questionnaire | Red meat | Prostate cancer | 65.3 g/d vs. never | 0.99 (0.81, 1.21) | 1, 4, 6, 10, 13, 16, 28 |
| Wright et al. (35) | Alpha-Tocopherol, Beta-Carotene Cancer Prevention Study (ATBC)/Finland | 50-69 | 27,111 (1,929) | 21 | Questionnaire | Beef | All prostate cancer | Q4 vs. Q1 | 0.97 (0.85, 1.10) | 1, 6, 9, 14, 35, 58 |
| Agalliu et al. (37) | Canadian Study of Diet, Lifestyle, and Health (CSDLH)/Canada | Cases:  66.2  Subcohort: 69.3 | Cases:  661  Subcohort: 1,864 | 8 | FFQ | Red meat | All prostate cancer | 1.3 vs. 0.7 oz | 1.44 (1.06, 1.95) | 1, 4, 9, 11, 18 |
| Sinha et al.  (6) | National Institutes of Health (NIH)-AARP Diet and Health Study/USA | 50-71 | 175,343 (10,313) | 9 | FFQ | Red meat | Total prostate cancer | 66.1 vs. 11.6 g/1,000 kcal | 1.12 (1.04, 1.21) | 1, 4, 6, 9, 12, 13, 16, 17, 18, 19, 22, 24, 25, 28, 31, 41, 43, 53 |
| Allen et al.  (38) | European Prospective Investigation into Cancer and Nutrition (EPIC)/10 European countries^*^ | 52 | 142,520 (2,441) | 8.7 | FFQ or diet histories | Red meat | Prostate cancer | Q5 vs. Q1 | 0.96 (0.82, 1.12) | 2, 3, 6, 9, 16 |
| Koutros et al. (39) | Agricultural Health Study/USA | 48.3 | 23,080  (668) | 10 | FFQ | Red meat | Prostate cancer | 122.3 vs. 23.2 g/d | 1.10 (0.85, 1.43) | 1, 13, 18, 21, 39 |
| Park et al.  (41) | Multiethnic Cohort Study/USA | ≥45 | 82,483 (4,404) | 8 | FFQ | Red meat | Total prostate cancer | 37 vs. 5.5 g/1,000 kcal | 0.97 (0.87, 1.07) | 4, 6, 9, 13, 19, 21, 33 |
| Rohrmann et al. (42) | CLUE II/USA | ≥35 | 3,892  (199) | 15 | FFQ | Beef and pork | Total prostate cancer | T3 vs. T1 | 1.16 (0.85, 1.58) | 1, 5, 6, 43, 59 |
| Rodriguez et al. (11) | Cancer Prevention Study II Nutrition Cohort/USA | 50-74 | 65,548  (5,113) | 9 | FFQ | Unprocessed red meat | All prostate cancer | ≥423 vs. 0-<137 g/week | 1.00 (0.91, 1.11) | 1, 4, 6, 9, 21, 22, 28 |
| Cross et al.  (44) | Prostate, Lung, Colorectal, and Ovarian (PLCO) Cancer Screening Trial/USA | 55-74 | 29,361 (1,338) | 11 | FFQ | Red meat | Prostate cancer | >146-845.4 vs. 0-43.5 g/d | 0.91 (0.73, 1.12) | 1, 4, 6, 11, 13, 18, 21, 28, 31, 32, 34, 36, 45 |
| Allen et al.  (45) | Life Span Study/Japan | 51 | 18,115  (193) | 16.9 | FFQ | Pork | Prostate cancer | Almost daily vs.  <2 times/week | 1.24 (0.61, 2.54) | 1, 9, 26, 37, 38 |
| Le Marchand et al. (48) | Hawaiian State Department of Health/USA | ≥18 | 8,881  (198) | 14 | Questionnaire | Pork, beef | Prostate cancer | Highest vs. lowest | 1.35 (1.01, 1.82) | 1, 19, 35 |
| Gann et al.  (49) | Physicians' Health Study/USA | Cases:  64.7  Controls:  64.7 | Cases:  120  Controls:  120 | 6 | FFQ | Beef, pork, or lamb | Prostate cancer | ≥5-6 times/week vs. ≤1-3 times/month | 2.51 (0.93, 6.74) | 1, 13 |
| **Advanced prostate cancer** | | | | | | | | | | |
| Wright et al. (35) | Alpha-Tocopherol, Beta-Carotene Cancer Prevention Study (ATBC)/Finland | 50-69 | 27,111  (438) | 21 | Questionnaire | Beef | Advanced prostate cancer | Q4 vs. Q1 | 0.89 (0.68, 1.16) | 1, 6, 9, 14, 35, 58 |
| Richman et al. (36) | Health Professionals Follow-Up Study (HPFS)/ US | 40-75 | 27,607  (199) | 22 | FFQ | Total unprocessed red meat | Lethal prostate cancer | ≥5 vs. <2  servings/week | 0.64 (0.38, 1.06) | 1, 4, 6, 12, 13, 45, 50 |
| Agalliu et al. (37) | Canadian Study of Diet, Lifestyle, and Health (CSDLH)/Canada | Cases:  66.2  Subcohort: 69.3 | Cases:  173  Subcohort: 1,864 | 8 | FFQ | Red meat | Advanced prostate cancer | 1.3 vs. 0.7 oz | 1.38 (0.80, 2.39) | 1, 4, 9, 11, 18 |
| Sinha et al.  (6) | National Institutes of Health (NIH)-AARP Diet and Health Study/USA | 50-71 | 175,343  (1,521) | 9 | FFQ | Red meat | Advanced  and fatal prostate cancer | 66.1 vs. 11.6 g/1,000 kcal | 1.29 (1.06, 1.56) | 1, 4, 6, 9, 12, 13, 16, 17, 18, 19, 22, 24, 25, 28, 31, 41, 43, 53 |
| Koutros et al. (39) | Agricultural Health Study/USA | 48.3 | 23,080  (140) | 10 | FFQ | Red meat | Advanced prostate cancer | 122.3 vs. 23.2 g/d | 0.89 (0.50, 1.60) | 1, 13, 18, 21, 39 |
| Park et al.  (41) | Multiethnic Cohort Study/USA | ≥45 | 82,483 (1,278) | 8 | FFQ | Red meat | Non-localized or high-grade cancer | 37 vs. 5.5 g/1,000 kcal | 0.95 (0.79, 1.14) | 4, 6, 9, 13, 19, 21, 33 |
| Rohrmann et al. (42) | CLUE II/USA | ≥35 | 3,892  (54) | 15 | FFQ | Beef and pork | High-stage prostate cancer | T3 vs. T1 | 1.25 (0.68, 2.30) | 1, 5, 6, 43, 59 |
| Rodriguez et al. (11) | Cancer Prevention Study II Nutrition Cohort/USA | 50-74 | 64,856  (225) | 9 | FFQ | Unprocessed red meat | Metastatic prostate cancer | ≥423 vs. 0-<137 g/week | 0.8 (0.5, 1.2) | 1, 4, 6, 9, 21, 22, 28 |
| Wu et al.  (43) | Health Professionals Follow-Up Study (HPFS)/USA | 40-75 | 47,725  (484) | 14 | FFQ | Total red meat | Advanced prostate cancer | Q5 vs. Q1 | 1.43 (1.08, 1.89) | 1, 2, 4, 6, 12, 13, 17, 18, 21, 27 |
| Cross et al.  (44) | Prostate, Lung, Colorectal, and Ovarian (PLCO) Cancer Screening Trial/USA | 55-74 | 29,361  (520) | 11 | FFQ | Red meat | Advanced prostate cancer | >146-845.4 vs. 0-43.5 g/d | 0.92 (0.66, 1.29) | 1, 4, 6, 11, 13, 18, 21, 28, 31, 32, 34, 36, 45 |
| **Processed meat consumption** | | | | | | | | | | |
| **Total prostate cancer** | | | | | | | | | | |
| Knuppel et al. (32) | National Health Service (NHS)/ England | 37-73 | 219,039 (5,958) | 6.9 | 24 h-dietary recall | Processed meat | Prostate cancer | Per 20 g/day | 1.00 (0.92, 1.08) | 1, 2, 4, 11, 13, 17, 19, 55, 56, 45, 60, 61, 62, 63, 64, 65, 66, 67 |
| Diallo et al.  (7) | NutriNet-Santé study/France | ≥35 | 15,546  (222) | 7 | 24 h-dietary records | Processed meat | Prostate cancer | 38.6±34.6 vs. 3.8±6.1 g/d | 1.35 (0.84, 2.20) | 1, 2, 4, 7, 8, 9, 11, 13, 17, 20, 40, 50, 55, 56, 57 |
| Lane et al.  (34) | EPIC-Norfolk, EPIC-Oxford, NSHD, Whitehall II, and ProtecT/UK | Cases:  63  Controls:  62.7 | Cases:  1,717  Controls:  3,528 | 6.6-13.3 | Questionnaire | Processed meat | Prostate cancer | 43.7 g/d vs. never | 1.14 (0.93, 1.39) | 1, 4, 6, 10, 13, 16, 28 |
| Wright et al. (35) | Alpha-Tocopherol, Beta-Carotene Cancer Prevention Study (ATBC)/Finland | 50-69 | 27,111 (1,929) | 21 | Questionnaire | Sausages | All prostate cancer | Q4 vs. Q1 | 1.08 (0.95, 1.23) | 1, 6, 9, 14, 35, 58 |
| Sinha et al.  (6) | National Institutes of Health (NIH)-AARP Diet and Health Study/USA | 50-71 | 175,343 (10,313) | 9 | FFQ | Processed meat | Total prostate cancer | 24.6 vs. 2.2 g/1,000 kcal | 1.07 (1.00, 1.14) | 1, 4, 6, 9, 12, 13, 16, 17, 18, 19, 22, 24, 25, 28, 31, 41, 43, 53 |
| Allen et al.  (38) | European Prospective Investigation into Cancer and Nutrition (EPIC)/10 European countries^*^ | 52 | 142,520 (2,727) | 8.7 | FFQ or diet histories | Processed meat | Prostate cancer | Q5 vs. Q1 | 0.93 (0.79, 1.09) | 2, 3, 6, 9, 16 |
| Koutros et al. (39) | Agricultural Health Study/USA | 48.3 | 23,080  (668) | 10 | FFQ | Bacon,  sausage,  hamburgers | Prostate cancer | Q5 vs. Q1 | 1.00 (0.82, 1.22) | 1, 13, 18, 21, 39 |
| Park et al.  (41) | Multiethnic Cohort Study/USA | ≥45 | 82,483 (4,404) | 8 | FFQ | Processed meat | Total prostate cancer | 20 vs. 2.2 g/1,000 kcal | 1.01 (0.91, 1.12) | 4, 6, 9, 13, 19, 21, 33 |
| Rohrmann et al. (42) | CLUE II/USA | ≥35 | 3,892  (199) | 15 | FFQ | Processed meat | Total prostate cancer | ≥5 vs. ≤1 time/week | 1.53 (0.98, 2.39) | 1, 5, 6, 43, 59 |
| Rodriguez et al. (11) | Cancer Prevention Study II Nutrition Cohort/USA | 50-74 | 65,548  (5,113) | 9 | FFQ | Processed meat | All prostate cancer | ≥247 vs. 0-<59 g/week | 1.01 (0.92, 1.12) | 1, 4, 6, 9, 21, 22, 28 |
| Cross et al.  (44) | Prostate, Lung, Colorectal, and Ovarian (PLCO) Cancer Screening Trial/USA | 55-74 | 29,361 (1,338) | 11 | FFQ | Processed meat | Prostate cancer | >36.8-367.1 vs. 0-6.7 g/d | 1.14 (0.93, 1.39) | 1, 4, 6, 11, 13, 18, 21, 28, 31, 32, 34, 36, 45 |
| Schuurman et al. (47) | The Netherlands Cohort Study (NLCS)/ Netherlands | 55-69 | Cases:  642  Subcohort:  1,525 | 6.3 | FFQ | Cured meat | Prostate cancer | 36 vs. 0 g/d | 1.37 (1.00, 1.89) | 1, 10, 21 |
| Le Marchand et al. (48) | Hawaiian State Department of Health/USA | ≥18 | 8,881  (198) | 14 | Questionnaire | Processed meat | Prostate cancer | Q4 vs. Q1 | 1.2 (0.8, 1.9) | 1, 19, 35 |
| Severson et al.  (52) | Hawaiian Men of Japanese Ancestry/USA | 46-65 | 7,998  (174) | 21 | FFQ | Ham, bacon, sausage | Prostate cancer | ≥5 vs. ≤1 time/week | 1.11 (0.75, 1.65) | 1 |
| **Advanced prostate cancer** | | | | | | | | | | |
| Kenfield et al.  (19) | Health Professionals Follow-up Study (HPFS) and The Physicians’ Health Study (PHS)/USA | 40-84 | 63,025  (913) | 23-24 | FFQ | Processed meat | Lethal prostate cancer | ≥3 vs. <3 servings/week | 1.29 (1.16, 1.42) | 1, 4, 12, 13, 18, 23, 28, 31, 42, 43 |
| Wright et al. (35) | Alpha-Tocopherol, Beta-Carotene Cancer Prevention Study (ATBC)/Finland | 50-69 | 27,111  (438) | 21 | Questionnaire | Sausages | Advanced prostate cancer | Q4 vs. Q1 | 1.02 (0.78, 1.35) | 1, 6, 9, 14, 35, 58 |
| Sinha et al.  (6) | National Institutes of Health (NIH)-AARP Diet and Health Study/USA | 50-71 | 175,343 (1,521) | 9 | FFQ | Processed meat | Advanced prostate cancer | 24.6 vs. 2.2 g/1,000 kcal | 1.16 (0.98, 1.38) | 1, 4, 6, 9, 12, 13, 16, 17, 18, 19, 22, 24, 25, 28, 31, 41, 43, 53 |
| Koutros et al. (39) | Agricultural Health Study/USA | 48.3 | 23,080  (140) | 10 | FFQ | Bacon,  sausage,  hamburgers | Advanced prostate cancer | Q5 vs. Q1 | 0.79 (0.50, 1.24) | 1, 13, 18, 21, 39 |
| Park et al.  (41) | Multiethnic Cohort Study/USA | ≥45 | 82,483 (1,278) | 8 | FFQ | Processed meat | Non-localized or high-grade cancer | 20 vs. 2.2 g/1,000 kcal | 0.92 (0.77, 1.11) | 4, 6, 9, 13, 19, 21, 33 |
| Rohrmann et al. (42) | CLUE II/USA | ≥35 | 3,892  (54) | 15 | FFQ | Processed meats | High-stage prostate cancer | ≥5 vs. ≤1 time/week | 2.24 (0.90, 5.59) | 1, 5, 6, 43, 59 |
| Rodriguez et al. (11) | Cancer Prevention Study II Nutrition Cohort/USA | 50-74 | 64,856  (225) | 9 | FFQ | Processed meat | Metastatic prostate cancer | ≥247 vs. 0-<59 g/week | 1.1 (0.7, 1.7) | 1, 4, 6, 9, 21, 22, 28 |
| Cross et al.  (44) | Prostate, Lung, Colorectal, and Ovarian (PLCO) Cancer Screening Trial/USA | 55-74 | 29,361  (520) | 11 | FFQ | Processed meat | Advanced prostate cancer | >36.8-367.1 vs. 0-6.7 g/d | 1.37 (0.99, 1.90) | 1, 4, 6, 11, 13, 18, 21, 28, 31, 32, 34, 36, 45 |
| **Red and processed meat consumption** | | | | | | | | | | |
| **Total prostate cancer** | | | | | | | | | | |
| Knuppel et al. (32) | National Health Service (NHS)/ England | 37-73 | 219,039 (5,958) | 6.9 | 24 h-dietary recall | Red and processed meat | Prostate cancer | Per 70 g/day | 1.04 (0.93, 1.17) | 1, 2, 4, 11, 13, 17, 19, 55, 56, 45, 60, 61, 62, 63, 64, 65, 66, 67 |
| Diallo et al.  (7) | NutriNet-Santé study/France | ≥35 | 15,546  (222) | 7 | 24 h-dietary records | Red and processed meat | Prostate cancer | Q5 vs. Q1 | 1.17 (0.72, 1.89) | 1, 2, 4, 7, 8, 9, 11, 13, 17, 20, 40, 50, 55, 56, 57 |
| Lane et al.  (34) | EPIC-Norfolk, EPIC-Oxford, NSHD, Whitehall II, and ProtecT/UK | Cases:  63  Controls:  62.7 | Cases:  1,717  Controls:  3,528 | 6.6-13.3 | Questionnaire | Red and processed meat | Prostate cancer | 99.7 g/d vs. never | 1.05 (0.86, 1.29) | 1, 4, 6, 10, 13, 16, 28 |
| Wright et al. (35) | Alpha-Tocopherol, Beta-Carotene Cancer Prevention Study (ATBC)/Finland | 50-69 | 27,111 (1,929) | 21 | Questionnaire | Red meat (beef and sausages) | All prostate cancer | Q4 vs. Q1 | 0.89 (0.78, 1.01) | 1, 6, 9, 14, 35, 58 |
| Sinha et al.  (6) | National Institutes of Health (NIH)-AARP Diet and Health Study/USA | 50-71 | 175,343 (10,313) | 9 | FFQ | Red and processed meat | Total prostate cancer | Q5 vs. Q1 | 1.09 (1.03, 1.14) | 1, 4, 6, 9, 12, 13, 16, 17, 18, 19, 22, 24, 25, 28, 31, 41, 43, 53 |
| Allen et al.  (38) | European Prospective Investigation into Cancer and Nutrition (EPIC)/10 European countries^*^ | 52 | 142,520 (2,727) | 8.7 | FFQ or diet histories | Red and processed meat | Prostate cancer | Q5 vs. Q1 | 0.94 (0.84, 1.05) | 2, 3, 6, 9, 16 |
| Koutros et al. (39) | Agricultural Health Study/USA | 48.3 | 23,080  (668) | 10 | FFQ | Red and processed meat | Prostate cancer | Q5 vs. Q1 | 1.03 (0.88, 1.21) | 1, 13, 18, 21, 39 |
| Park et al.  (41) | Multiethnic Cohort Study/USA | ≥45 | 82,483 (4,404) | 8 | FFQ | Red and processed meat | Total prostate cancer | Q5 vs. Q1 | 0.99 (0.92, 1.06) | 4, 6, 9, 13, 19, 21, 33 |
| Rohrmann et al. (42) | CLUE II/USA | ≥35 | 3,892  (199) | 15 | FFQ | Red meat (red and processed meat) | Total prostate cancer | T3 vs. T1 | 0.87 (0.59, 1.32) | 1, 5, 6, 43, 59 |
| Rodriguez et al. (11) | Cancer Prevention Study II Nutrition Cohort/USA | 50-74 | 65,548  (5,113) | 9 | FFQ | Total processed and unprocessed  red meat | All prostate cancer | ≥657 vs. 0-<246 g/week | 1.00 (0.95, 1.05) | 1, 4, 6, 9, 21, 22, 28 |
| Cross et al.  (44) | Prostate, Lung, Colorectal, and Ovarian (PLCO) Cancer Screening Trial/USA | 55-74 | 29,361 (1,338) | 11 | FFQ | Red and processed meat | Prostate cancer | Q5 vs. Q1 | 1.02 (0.88, 1.18) | 1, 4, 6, 11, 13, 18, 21, 28, 31, 32, 34, 36, 45 |
| Michaud et al.  (46) | Health Professionals Follow-Up Study (HPFS)/USA | 40-75 | 47,780  (1,897) | 10 | FFQ | Red meat (red and processed meat) | Prostate cancer | Q5 vs. Q1 | 0.91 (0.75, 1.1) | 1, 6, 12, 13, 41, 43, 53, 59 |
| Le Marchand et al. (48) | Hawaiian State Department of Health/USA | ≥18 | 8,881  (198) | 14 | Questionnaire | Red and processed meat | Prostate cancer | Highest vs. lowest | 1.30 (1.02, 1.65) | 1, 19, 35 |
| Mills et al.  (51) | Adventist Health Study/USA | ≥25 | 14,000  (180) | 6 | FFQ | Beef hamburger, beef steak, and other beef and  veal | Prostate cancer | ≥1 times/week vs. never | 0.96 (0.77, 1.21) | 1 |
| **Advanced prostate cancer** | | | | | | | | | | |
| Wright et al. (35) | Alpha-Tocopherol, Beta-Carotene Cancer Prevention Study (ATBC)/Finland | 50-69 | 27,111  (438) | 21 | Questionnaire | Red meat (beef and sausages) | Advanced prostate cancer | Q4 vs. Q1 | 0.80 (0.62, 1.05) | 1, 6, 9, 14, 35, 58 |
| Richman et al. (36) | Health Professionals Follow-Up Study (HPFS)/ US | 40-75 | 27,607  (199) | 22 | FFQ | Total red meat | Lethal prostate cancer | ≥8 vs. <3 servings/week | 1.07 (0.66, 1.75) | 1, 4, 6, 12, 13, 45, 51 |
| Sinha et al.  (6) | National Institutes of Health (NIH)-AARP Diet and Health Study/USA | 50-71 | 175,343  (1,521) | 9 | FFQ | Red and processed meat | Advanced  prostate cancer | Q5 vs. Q1 | 1.21 (1.06, 1.38) | 1, 4, 6, 9, 12, 13, 16, 17, 18, 19, 22, 24, 25, 28, 31, 41, 43, 53 |
| Koutros et al. (39) | Agricultural Health Study/USA | 48.3 | 23,080  (140) | 10 | FFQ | Red and processed meat | Advanced prostate cancer | Q5 vs. Q1 | 0.82 (0.57, 1.18) | 1, 13, 18, 21, 39 |
| Park et al.  (41) | Multiethnic Cohort Study/USA | ≥45 | 82,483 (1,278) | 8 | FFQ | Red and processed meat | Non-localized or high-grade cancer | Q5 vs. Q1 | 0.93 (0.82, 1.06) | 4, 6, 9, 13, 19, 21, 33 |
| Rohrmann et al. (42) | CLUE II/USA | ≥35 | 3,892  (54) | 15 | FFQ | Red meat (red and processed meat) | High-stage prostate cancer | T3 vs. T1 | 0.87 (0.39, 1.93) | 1, 5, 6, 43, 59 |
| Rodriguez et al. (11) | Cancer Prevention Study II Nutrition Cohort/USA | 50-74 | 64,856  (225) | 9 | FFQ | Total processed and unprocessed red meat | Metastatic prostate cancer | ≥657 vs. 0-<246 g/week | 0.8 (0.5, 1.3) | 1, 4, 6, 9, 21, 22, 28 |
| Wu et al.  (43) | Health Professionals Follow-Up Study (HPFS)/USA | 40-75 | 47,725  (484) | 14 | FFQ | Red and processed meat | Advanced prostate cancer | Q5 vs. Q1 | 1.33 (1.08, 1.63) | 1, 2, 4, 6, 12, 13, 17, 18, 21, 27 |
| Cross et al.  (44) | Prostate, Lung, Colorectal, and Ovarian (PLCO) Cancer Screening Trial/USA | 55-74 | 29,361  (520) | 11 | FFQ | Red and processed meat | Advanced prostate cancer | Q5 vs. Q1 | 1.12 (0.89, 1.42) | 1, 4, 6, 11, 13, 18, 21, 28, 31, 32, 34, 36, 45 |
| Hsing et al.  (50) | Lutheran  Brotherhood Cohort/USA | ≥35 | 17,633  (149) | 20 | Questionnaire | Beef, bacon,  fresh pork, smoked ham | Fatal prostate cancer | >39 vs. <17 times/month | 0.8 (0.5, 1.3) | 1, 15 |
| **Total meat consumption** | | | | | | | | | | |
| **Total prostate cancer** | | | | | | | | | | |
| Knuppel et al. (32) | National Health Service (NHS)/ England | 37-73 | 219,039 (5,958) | 6.9 | 24 h-dietary recall | Red and processed meat | Prostate cancer | Per 70 g/day | 1.04 (0.93, 1.17) | 1, 2, 4, 11, 13, 17, 19, 55, 56, 45, 60, 61, 62, 63, 64, 65, 66, 67 |
| Kim et al.  (33) | National Health Insurance Corporation (NHIC)/Korea | 30-80 | 1,179,172  (2,747) | 8 | Questionnaire | Meat | Prostate cancer | ≥4 vs. ≤1 time/week | 1.17 ( 1.01, 1.35) | 1, 2, 4, 11, 13, 17, 20, 29, 48 |
| Diallo et al.  (7) | NutriNet-Santé study/France | ≥35 | 15,546  (222) | 7 | 24 h-dietary records | Red and processed meat | Prostate cancer | Q5 vs. Q1 | 1.17 (0.72, 1.89) | 1, 2, 4, 7, 8, 9, 11, 13, 17, 20, 40, 50, 55, 56, 57 |
| Lane et al.  (34) | EPIC-Norfolk, EPIC-Oxford, NSHD, Whitehall II, and ProtecT/UK | Cases:  63  Controls:  62.7 | Cases:  1,717  Controls:  3,528 | 6.6-13.3 | Questionnaire | Red and processed meat | Prostate cancer | 99.7 g/d vs. never | 1.05 (0.86, 1.29) | 1, 4, 6, 10, 13, 16, 28 |
| Wright et al. (35) | Alpha-Tocopherol, Beta-Carotene Cancer Prevention Study (ATBC)/Finland | 50-69 | 27,111 (1,929) | 21 | Questionnaire | Red meat (beef and sausages) | All prostate cancer | Q4 vs. Q1 | 0.89 (0.78, 1.01) | 1, 6, 9, 14, 35, 58 |
| Agalliu et al. (37) | Canadian Study of Diet, Lifestyle, and Health (CSDLH)/Canada | Cases:  66.2  Subcohort: 69.3 | Cases:  661  Subcohort: 1,864 | 8 | FFQ | Red meat | All prostate cancer | Q5 vs. Q1 | 1.44 (1.06, 1.95) | 1, 4, 9, 11, 18 |
| Sinha et al.  (6) | National Institutes of Health (NIH)-AARP Diet and Health Study/USA | 50-71 | 175,343 (10,313) | 9 | FFQ | Red and processed meat | Total prostate cancer | Q5 vs. Q1 | 1.09 (1.03, 1.14) | 1, 4, 6, 9, 12, 13, 16, 17, 18, 19, 22, 24, 25, 28, 31, 41, 43, 53 |
| Allen et al.  (38) | European Prospective Investigation into Cancer and Nutrition (EPIC)/10 European countries^*^ | 52 | 142,520 (2,727) | 8.7 | FFQ or diet histories | Red and processed meat | Prostate cancer | Q5 vs. Q1 | 0.94 (0.84, 1.05) | 2, 3, 6, 9, 16 |
| Koutros et al. (39) | Agricultural Health Study/USA | 48.3 | 23,080  (668) | 10 | FFQ | Red and processed meat | Prostate cancer | Q5 vs. Q1 | 1.03 (0.88, 1.21) | 1, 13, 18, 21, 39 |
| Park et al.  (41) | Multiethnic Cohort Study/USA | ≥45 | 82,483 (4,404) | 8 | FFQ | Red and processed meat | Total prostate cancer | Q5 vs. Q1 | 0.99 (0.92, 1.06) | 4, 6, 9, 13, 19, 21, 33 |
| Rohrmann et al. (42) | CLUE II/USA | ≥35 | 3,892  (199) | 15 | FFQ | Red meat (red and processed meat) | Total prostate cancer | T3 vs. T1 | 0.87 (0.59, 1.32) | 1, 5, 6, 43, 59 |
| Neuhouser et al. (40) | Carotene and Retinol Efficacy Trial (CARET)/USA | 50-69 | 12,000  (811) | 11 | FFQ | Total meat | Prostate cancer | ≥1.3 vs. <0.6 servings/d | 0.95 (0.75, 1.20) | 1, 4, 6, 13, 21 |
| Rodriguez et al. (11) | Cancer Prevention Study II Nutrition Cohort/USA | 50-74 | 65,548  (5,113) | 9 | FFQ | Total processed and unprocessed  red meat | All prostate cancer | ≥657 vs. 0-<246 g/week | 1.00 (0.95, 1.05) | 1, 4, 6, 9, 21, 22, 28 |
| Cross et al.  (44) | Prostate, Lung, Colorectal, and Ovarian (PLCO) Cancer Screening Trial/USA | 55-74 | 29,361 (1,338) | 11 | FFQ | Red and processed meat | Prostate cancer | Q5 vs. Q1 | 1.02 (0.88, 1.18) | 1, 4, 6, 11, 13, 18, 21, 28, 31, 32, 34, 36, 45 |
| Allen et al.  (45) | Life Span Study/Japan | 51 | 18,115  (193) | 16.9 | FFQ | Pork | Prostate cancer | Almost daily vs.  <2 times/week | 1.24 (0.61, 2.54) | 1, 9, 26, 37, 38 |
| Michaud et al.  (46) | Health Professionals Follow-Up Study (HPFS)/USA | 40-75 | 47,780  (1,897) | 10 | FFQ | Red meat (red and processed meat) | Prostate cancer | Q5 vs. Q1 | 0.91 (0.75, 1.1) | 1, 6, 12, 13, 41, 43, 53, 59 |
| Schuurman et al. (47) | The Netherlands Cohort Study (NLCS)/ Netherlands | 55-69 | Cases:  642  Subcohort:  1,525 | 6.3 | FFQ | Cured meat | Prostate cancer | 36 vs. 0 g/d | 1.37 (1.00, 1.89) | 1, 10, 21 |
| Le Marchand et al. (48) | Hawaiian State Department of Health/USA | ≥18 | 8,881  (198) | 14 | Questionnaire | Red and processed meat | Prostate cancer | Highest vs. lowest | 1.30 (1.02, 1.65) | 1, 19, 35 |
| Gann et al.  (49) | Physicians' Health Study/USA | Cases:  64.7  Controls:  64.7 | Cases:  120  Controls:  120 | 6 | FFQ | Beef, pork, or lamb | Prostate cancer | ≥5-6 times/week vs. ≤1-3 times/month | 2.51 (0.93, 6.74) | 1, 13 |
| Mills et al.  (51) | Adventist Health Study/USA | ≥25 | 14,000  (180) | 6 | FFQ | Beef hamburger, beef steak, and other beef and  veal | Prostate cancer | ≥1 times/week vs. never | 0.96 (0.77, 1.21) | 1 |
| Severson et al.  (52) | Hawaiian Men of Japanese Ancestry/USA | 46-65 | 7,999  (174) | 21 | FFQ | Meat | Prostate cancer | ≥5 vs. ≤1 time/week | 0.95 (0.61, 1.49) | 1 |
| **Advanced prostate cancer** | | | | | | | | | | |
| Kenfield et al.  (19) | The Physicians’ Health Study (PHS)/USA | 40-84 | 20,324  (337) | 23 | FFQ | Processed meat | Lethal prostate cancer | ≥3 vs. <3 servings/week | 1.3 (1.15, 1.54) | 1, 4, 12, 13, 18, 23, 28, 31, 42, 43 |
| Wright et al. (35) | Alpha-Tocopherol, Beta-Carotene Cancer Prevention Study (ATBC)/Finland | 50-69 | 27,111  (438) | 21 | Questionnaire | Red meat (beef and sausages) | Advanced prostate cancer | Q4 vs. Q1 | 0.80 (0.62, 1.05) | 1, 6, 9, 14, 35, 58 |
| Richman et al. (36) | Health Professionals Follow-Up Study (HPFS)/ US | 40-75 | 27,607  (199) | 22 | FFQ | Total red meat | Lethal prostate cancer | ≥8 vs. <3 servings/week | 1.07 (0.66, 1.75) | 1, 4, 6, 12, 13, 45, 51 |
| Agalliu et al. (37) | Canadian Study of Diet, Lifestyle, and Health (CSDLH)/Canada | Cases:  66.2  Subcohort: 69.3 | Cases:  173  Subcohort: 1,864 | 8 | FFQ | Red meat | Advanced prostate cancer | Q5 vs. Q1 | 1.38 (0.80, 2.39) | 1, 4, 9, 11, 18 |
| Sinha et al.  (6) | National Institutes of Health (NIH)-AARP Diet and Health Study/USA | 50-71 | 175,343  (1,521) | 9 | FFQ | Red and processed meat | Advanced  prostate cancer | Q5 vs. Q1 | 1.21 (1.06, 1.38) | 1, 4, 6, 9, 12, 13, 16, 17, 18, 19, 22, 24, 25, 28, 31, 41, 43, 53 |
| Koutros et al. (39) | Agricultural Health Study/USA | 48.3 | 23,080  (140) | 10 | FFQ | Red and processed meat | Advanced prostate cancer | Q5 vs. Q1 | 0.82 (0.57, 1.18) | 1, 13, 18, 21, 39 |
| Park et al.  (41) | Multiethnic Cohort Study/USA | ≥45 | 82,483 (1,278) | 8 | FFQ | Red and processed meat | Non-localized or high-grade cancer | Q5 vs. Q1 | 0.93 (0.82, 1.06) | 4, 6, 9, 13, 19, 21, 33 |
| Rohrmann et al. (42) | CLUE II/USA | ≥35 | 3,892  (54) | 15 | FFQ | Red meat (red and processed meat) | High-stage prostate cancer | T3 vs. T1 | 0.87 (0.39, 1.93) | 1, 5, 6, 43, 59 |
| Neuhouser et al. (40) | Carotene and Retinol Efficacy Trial (CARET)/USA | 50-69 | 12,000  (279) | 11 | FFQ | Total meat | Aggressive prostate cancer (stage III/IV) | ≥1.3 vs. <0.6 servings/d | 0.93 (0.62, 1.40) | 1, 4, 6, 13, 21 |
| Rodriguez et al. (11) | Cancer Prevention Study II Nutrition Cohort/USA | 50-74 | 64,856  (225) | 9 | FFQ | Total processed and unprocessed red meat | Metastatic prostate cancer | ≥657 vs. 0-<246 g/week | 0.8 (0.5, 1.3) | 1, 4, 6, 9, 21, 22, 28 |
| Wu et al.  (43) | Health Professionals Follow-Up Study (HPFS)/USA | 40-75 | 47,725  (484) | 14 | FFQ | Red and processed meat | Advanced prostate cancer | Q5 vs. Q1 | 1.33 (1.08, 1.63) | 1, 2, 4, 6, 12, 13, 17, 18, 21, 27 |
| Cross et al.  (44) | Prostate, Lung, Colorectal, and Ovarian (PLCO) Cancer Screening Trial/USA | 55-74 | 29,361  (520) | 11 | FFQ | Red and processed meat | Advanced prostate cancer | Q5 vs. Q1 | 1.12 (0.89, 1.42) | 1, 4, 6, 11, 13, 18, 21, 28, 31, 32, 34, 36, 45 |
| Hsing et al.  (50) | Lutheran  Brotherhood Cohort/USA | ≥35 | 17,633  (149) | 20 | Questionnaire | Beef, bacon,  fresh pork, smoked ham | Fatal prostate cancer | >39 vs. <17 times/month | 0.8 (0.5, 1.3) | 1, 15 |
| **All outcomes of prostate cancer** | | | | | | | | | | |
| Knuppel et al. (32) | National Health Service (NHS)/ England | 37-73 | 219,039 (5,958) | 6.9 | 24 h-dietary recall | Red and processed meat | Prostate cancer | Per 70 g/day | 1.04 (0.93, 1.17) | 1, 2, 4, 11, 13, 17, 19, 55, 56, 45, 60, 61, 62, 63, 64, 65, 66, 67 |
| Kim et al.  (33) | National Health Insurance Corporation (NHIC)/Korea | 30-80 | 1,179,172  (2,747) | 8 | Questionnaire | Meat | Prostate cancer | ≥4 vs. ≤1 time/week | 1.17 ( 1.01, 1.35) | 1, 2, 4, 11, 13, 17, 20, 29, 48 |
| Diallo et al.  (7) | NutriNet-Santé study/France | ≥35 | 15,546  (222) | 7 | 24 h-dietary records | Red and processed meat | Prostate cancer | Q5 vs. Q1 | 1.17 (0.72, 1.89) | 1, 2, 4, 7, 8, 9, 11, 13, 17, 20, 40, 50, 55, 56, 57 |
| Lane et al.  (34) | EPIC-Norfolk, EPIC-Oxford, NSHD, Whitehall II, and ProtecT/UK | Cases:  63  Controls:  62.7 | Cases:  1,717  Controls:  3,528 | 6.6-13.3 | Questionnaire | Red and processed meat | Prostate cancer | 99.7 g/d vs. never | 1.05 (0.86, 1.29) | 1, 4, 6, 10, 13, 16, 28 |
| Kenfield et al.  (19) | The Physicians’ Health Study (PHS)/USA | 40-84 | 20,324  (337) | 23 | FFQ | Processed meat | Lethal prostate cancer | ≥3 vs. <3 servings/week | 1.3 (1.15, 1.54) | 1, 4, 12, 13, 18, 23, 28, 31, 42, 43 |
| Wright et al. (35) | Alpha-Tocopherol, Beta-Carotene Cancer Prevention Study (ATBC)/Finland | 50-69 | 27,111 (1,929) | 21 | Questionnaire | Red meat (beef and sausages) | All prostate cancer | Q4 vs. Q1 | 0.89 (0.78, 1.01) | 1, 6, 9, 14, 35, 58 |
| Richman et al. (36) | Health Professionals Follow-Up Study (HPFS)/ US | 40-75 | 27,607  (199) | 22 | FFQ | Total red meat | Lethal prostate cancer | ≥8 vs. <3 servings/week | 1.07 (0.66, 1.75) | 1, 4, 6, 12, 13, 45, 51 |
| Agalliu et al. (37) | Canadian Study of Diet, Lifestyle, and Health (CSDLH)/Canada | Cases:  66.2  Subcohort: 69.3 | Cases:  661  Subcohort: 1,864 | 8 | FFQ | Red meat | All prostate cancer | Q5 vs. Q1 | 1.44 (1.06, 1.95) | 1, 4, 9, 11, 18 |
| Sinha et al.  (6) | National Institutes of Health (NIH)-AARP Diet and Health Study/USA | 50-71 | 175,343 (10,313) | 9 | FFQ | Red and processed meat | Total prostate cancer | Q5 vs. Q1 | 1.09 (1.03, 1.14) | 1, 4, 6, 9, 12, 13, 16, 17, 18, 19, 22, 24, 25, 28, 31, 41, 43, 53 |
| Allen et al.  (38) | European Prospective Investigation into Cancer and Nutrition (EPIC)/10 European countries^*^ | 52 | 142,520 (2,727) | 8.7 | FFQ or diet histories | Red and processed meat | Prostate cancer | Q5 vs. Q1 | 0.94 (0.84, 1.05) | 2, 3, 6, 9, 16 |
| Koutros et al. (39) | Agricultural Health Study/USA | 48.3 | 23,080  (668) | 10 | FFQ | Red and processed meat | Prostate cancer | Q5 vs. Q1 | 1.03 (0.88, 1.21) | 1, 13, 18, 21, 39 |
| Park et al.  (41) | Multiethnic Cohort Study/USA | ≥45 | 82,483 (4,404) | 8 | FFQ | Red and processed meat | Total prostate cancer | Q5 vs. Q1 | 0.99 (0.92, 1.06) | 4, 6, 9, 13, 19, 21, 33 |
| Rohrmann et al. (42) | CLUE II/USA | ≥35 | 3,892  (199) | 15 | FFQ | Red meat ( red and processed meat ) | Total prostate cancer | T3 vs. T1 | 0.87 (0.59, 1.32) | 1, 5, 6, 43, 59 |
| Neuhouser et al. (40) | Carotene and Retinol Efficacy Trial (CARET)/USA | 50-69 | 12,000  (811) | 11 | FFQ | Total meat | Prostate cancer | ≥1.3 vs. <0.6 servings/d | 0.95 (0.75, 1.20) | 1, 4, 6, 13, 21 |
| Rodriguez et al. (11) | Cancer Prevention Study II Nutrition Cohort/USA | 50-74 | 65,548  (5,113) | 9 | FFQ | Total processed and unprocessed red meat | All prostate cancer | ≥657 vs. 0-<246 g/week | 1.00 (0.95, 1.05) | 1, 4, 6, 9, 21, 22, 28 |
| Wu et al.  (43) | Health Professionals Follow-Up Study (HPFS)/USA | 40-75 | 47,725  (484) | 14 | FFQ | Red and processed meat | Advanced prostate cancer | Q5 vs. Q1 | 1.33 (1.08, 1.63) | 1, 2, 4, 6, 12, 13, 17, 18, 21, 27 |
| Cross et al.  (44) | Prostate, Lung, Colorectal, and Ovarian (PLCO) Cancer Screening Trial/USA | 55-74 | 29,361 (1,338) | 11 | FFQ | Red and processed meat | Prostate cancer | Q5 vs. Q1 | 1.02 (0.88, 1.18) | 1, 4, 6, 11, 13, 18, 21, 28, 31, 32, 34, 36, 45 |
| Allen et al.  (45) | Life Span Study/Japan | 51 | 18,115  (193) | 16.9 | FFQ | Pork | Prostate cancer | Almost daily vs.  <2 times/week | 1.24 (0.61, 2.54) | 1, 9, 26, 37, 38 |
| Schuurman et al. (47) | The Netherlands Cohort Study (NLCS)/  Netherlands | 55-69 | Cases:  642  Subcohort:  1,525 | 6.3 | FFQ | Cured meat | Prostate cancer | 36 vs. 0 g/d | 1.37 (1.00, 1.89) | 1, 10, 21 |
| Le Marchand et al. (48) | Hawaiian State Department of Health/USA | ≥18 | 8,881  (198) | 14 | Questionnaire | Red and processed meat | Prostate cancer | Highest vs. lowest | 1.30 (1.02, 1.65) | 1, 19, 35 |
| Gann et al.  (49) | Physicians' Health Study/USA | Cases:  64.7  Controls:  64.7 | Cases:  120  Controls:  120 | 6 | FFQ | Beef, pork, or lamb | Prostate cancer | ≥5-6 times/week vs. ≤1-3 times/month | 2.51 (0.93, 6.74) | 1, 13 |
| Hsing et al.  (50) | Lutheran  Brotherhood Cohort/USA | ≥35 | 17,633  (149) | 20 | Questionnaire | Beef, bacon,  fresh pork, smoked ham | Fatal prostate cancer | >39 vs. <17 times/month | 0.8 (0.5, 1.3) | 1, 15 |
| Mills et al.  (51) | Adventist Health Study/USA | ≥25 | 14,000  (180) | 6 | FFQ | Beef hamburger, beef steak, and other beef and  veal | Prostate cancer | ≥1 times/week vs. never | 0.96 (0.77, 1.21) | 1 |
| Severson et al.  (52) | Hawaiian Men of Japanese Ancestry/USA | 46-65 | 7,999  (174) | 21 | FFQ | Meat | Prostate cancer | ≥5 vs. ≤1 time/week | 0.95 (0.61, 1.49) | 1 |

FFQ: food frequency questionnaire; g: grams; d: day; oz: ounce; RR: relative risk; CI: confidence interval.

Adjustments: Age = 1, Height = 2, Weight = 3, Body mass index = 4, BMI at age 21 = 5, Energy intake = 6, Energy intake without alcohol = 7, Sex = 8, Educational level = 9, Socioeconomic status = 10, Physical activity, exercise activity = 11, Vigorous physical activity = 12, Smoking = 13, Number of years as a smoker, smoking dose and duration = 14, Tobacco use = 15, Marital status = 16, Alcohol consumption = 17, Race = 18, Ethnicity = 19, Family history of cancers = 20, Family history of prostate cancer = 21, History of PSA testing, undergoing prostate-specific antigen testing in the past 3 years = 22, Random assignment status in the PHS = 23, Intakes of zinc = 24, Intakes of selenium = 25, Radiation dose = 26, History of vasectomy = 27, History of diabetes, diabetes = 28, Glucose levels = 29, Clinical stage = 30, Vitamin E use = 31, Aspirin use = 32, Time on study = 33, Study center = 34, Income by proportional hazards regression = 35, Number of screening exams during follow-up = 36, Calendar period = 37, City of residence = 38, State of residence = 39, Number of 24 h-dietary records = 40, Intakes of alpha-linolenic acid = 41, Fatty fish intake = 42, Supplementation group = 43, Consumption of tomato products, tomato intake= 43, Cooked tomato products intake = 44, Lycopene intake = 45, Consumption of total fresh meat and poultry = 46, Consumption of total cured meat = 47, The frequency of meat consumption = 48, Red meat intake = 49, Processed meat intake, Total processed red meat = 50, Eggs = 51, Unprocessed red meat = 52, Total calcium intake = 53, Coffee intake = 54, Fruit intakes = 55, Vegetables intake = 56, Lipids intake = 57, Dietary fat = 58, Intake of saturated fat = 59, Estimated cereal ﬁber intake = 60, Cheese intake = 61, Milk added to tea/coffee/cereal = 62, Non-oily ﬁsh intake = 63, Deprivation = 64, Qualiﬁcation = 65, Employment = 66, Living with a spouse or partner = 67, Fiber intake = 68, Dairy intake = 69.

^*^Including Denmark, France, Germany, Greece, Italy, the Netherlands, Norway, Spain, Sweden, and the United Kingdom.
