# Supplementary material for: Association Between Red and Processed Meat Consumption and Risk of Prostate Cancer: A Systematic Review and Meta-Analysis
Source: Front Nutr. 2022 Feb 7;9:801722. doi: 10.3389/fnut.2022.801722 (PMC8859108; doi:10.3389/fnut.2022.801722)
Supplement: Supplementary Table 2 — Results of subgroup analyses on the association of meat consumption and risk of prostate cancer. [file Table_2.DOCX]

| Supplementary Table 2. Results of subgroup analyses on the association of meat consumption and risk of prostate cancer | | | | | |
| --- | --- | --- | --- | --- | --- |
|  | ***No*. of studies** | **RR (95% CI)** | ***P*-within** | ***I^2^* (%)** | ***P*-between** |
| Subgroup analyses for red meat intake and risk of total prostate cancer | | | | | |
| US vs. Non-US |  |  |  |  | 0.408 |
| US | 8 | 1.05 (1.00, 1.11) | 0.05 | 49.9 |  |
| Non-US | 6 | 1.01 (0.93, 1.10) | 0.20 | 30.7 |  |
| Adjustment for energy intake |  |  |  |  | 0.006 |
| Yes | 9 | 1.03 (0.98, 1.07) | 0.222 | 24.9 |  |
| No | 5 | 1.29 (1.10, 1.51) | 0.437 | 0.0 |  |
| Adjustment for smoking |  |  |  |  | 0.958 |
| Yes | 8 | 1.04 (0.99, 1.10) | 0.09 | 42.4 |  |
| No | 6 | 1.04 (0.97, 1.13) | 0.08 | 48.5 |  |
| Adjustment for alcohol consumption |  |  |  |  | 0.018 |
| Yes | 2 | 1.12 (1.04, 1.21) | 0.60 | 0.0 |  |
| No | 12 | 1.01 (0.96, 1.06) | 0.14 | 31.4 |  |
| Adjustment for family history of cancer |  |  |  |  | 0.040 |
| Yes | 5 | 0.99 (0.93, 1.06) | 0.65 | 0.0 |  |
| No | 9 | 1.08 (1.02, 1.14) | 0.05 | 47.5 |  |
| Study quality |  |  |  |  | 0.068 |
| NOS score ≥7 | 6 | 0.99 (0.93, 1.06) | 0.73 | 0.0 |  |
| NOS score <7 | 8 | 1.08 (1.02, 1.13) | 0.02 | 55.6 |  |
| Subgroup analyses for red meat intake and risk of advanced prostate cancer | | | | | |
| US vs. Non-US |  |  |  |  | 0.353 |
| US | 7 | 1.10 (0.99, 1.22) | 0.05 | 51.0 |  |
| Non-US | 2 | 0.97 (0.76, 1.23) | 0.15 | 49.8 |  |
| Adjustment for energy intake |  |  |  |  | 0.823 |
| Yes | 7 | 1.07 (0.97, 1.18) | 0.03 | 56.8 |  |
| No | 2 | 1.12 (0.75, 1.67) | 0.28 | 13.7 |  |
| Adjustment for smoking |  |  |  |  | 0.854 |
| Yes | 6 | 1.08 (0.97, 1.19) | 0.03 | 59.4 |  |
| No | 3 | 1.05 (0.78, 1.41) | 0.25 | 27.4 |  |
| Adjustment for alcohol consumption |  |  |  |  | 0.001 |
| Yes | 2 | 1.33 (1.14, 1.56) | 0.55 | 0.0 |  |
| No | 7 | 0.95 (0.84, 1.07) | 0.74 | 0.0 |  |
| Adjustment for family history of cancer |  |  |  |  | 0.181 |
| Yes | 5 | 1.01 (0.89, 1.15) | 0.09 | 49.5 |  |
| No | 4 | 1.16 (1.00, 1.34) | 0.14 | 44.3 |  |
| Study quality |  |  |  |  | 0.560 |
| NOS score ≥7 | 5 | 1.04 (0.89, 1.21) | 0.07 | 53.1 |  |
| NOS score <7 | 4 | 1.10 (0.97, 1.25) | 0.10 | 51.9 |  |
| Subgroup analyses for processed meat intake and risk of total prostate cancer | | | | | |
| US vs. Non-US |  |  |  |  | 0.701 |
| US | 8 | 1.05 (1.01, 1.10) | 0.59 | 0.0 |  |
| Non-US | 5 | 1.07 (0.98, 1.17) | 0.16 | 38.7 |  |
| Adjustment for energy intake |  |  |  |  | 0.510 |
| Yes | 9 | 1.05 (1.01, 1.10) | 0.35 | 10.0 |  |
| No | 4 | 1.11 (0.96, 1.28) | 0.41 | 0.0 |  |
| Adjustment for smoking |  |  |  |  | 0.483 |
| Yes | 7 | 1.06 (1.02, 1.12) | 0.77 | 0.0 |  |
| No | 6 | 1.03 (0.95, 1.11) | 0.13 | 40.7 |  |
| Adjustment for alcohol consumption |  |  |  |  | 0.495 |
| Yes | 2 | 1.07 (1.01, 1.15) | 0.34 | 0.0 |  |
| No | 11 | 1.04 (0.99, 1.10) | 0.37 | 7.8 |  |
| Adjustment for family history of cancer |  |  |  |  | 0.480 |
| Yes | 6 | 1.04 (0.98, 1.10) | 0.34 | 10.7 |  |
| No | 7 | 1.07 (1.01, 1.12) | 0.41 | 1.5 |  |
| Study quality |  |  |  |  | 0.528 |
| NOS score ≥7 | 7 | 1.07 (1.00, 1.15) | 0.48 | 0.0 |  |
| NOS score <7 | 6 | 1.05 (0.99, 1.10) | 0.27 | 20.5 |  |
| Subgroup analyses for processed meat intake and risk of advanced prostate cancer | | | | | |
| US vs. Non-US |  |  |  |  | 0.305 |
| US | 7 | 1.18 (1.10, 1.27) | 0.01 | 62.3 |  |
| Non-US | 1 | 1.02 (0.78, 1.34) | . | . |  |
| Adjustment for energy intake |  |  |  |  | 0.036 |
| Yes | 6 | 1.08 (0.97, 1.20) | 0.14 | 39.7 |  |
| No | 2 | 1.26 (1.14, 1.39) | 0.03 | 76.6 |  |
| Adjustment for smoking |  |  |  |  | 0.717 |
| Yes | 6 | 1.17 (1.09, 1.26) | 0.01 | 66.6 |  |
| No | 2 | 1.26 (0.85, 1.88) | 0.17 | 46.9 |  |
| Adjustment for alcohol consumption |  |  |  |  | 0.899 |
| Yes | 1 | 1.16 (0.98, 1.38) | . | . |  |
| No | 7 | 1.17 (1.09, 1.27) | 0.009 | 64.6 |  |
| Adjustment for family history of cancer |  |  |  |  | 0.01 |
| Yes | 4 | 1.24 (1.14, 1.34) | 0.19 | 36.5 |  |
| No | 4 | 1.00 (0.86, 1.15) | 0.13 | 46.3 |  |
| Study quality |  |  |  |  | 0.974 |
| NOS score ≥7 | 4 | 1.18 (0.98, 1.42) | 0.27 | 22.6 |  |
| NOS score <7 | 4 | 1.17 (1.08, 1.27) | 0.004 | 77.1 |  |
| Subgroup analyses for red and processed meat intake and risk of total prostate cancer | | | | | |
| US vs. Non-US |  |  |  |  | 0.034 |
| US | 9 | 1.03 (1.00, 1.06) | 0.09 | 41.2 |  |
| Non-US | 4 | 0.94 (0.87, 1.02) | 0.45 | 0.0 |  |
| Adjustment for energy intake |  |  |  |  | 0.416 |
| Yes | 10 | 1.02 (0.99, 1.04) | 0.05 | 43.5 |  |
| No | 3 | 1.07 (0.95, 1.20) | 0.16 | 44.7 |  |
| Adjustment for smoking |  |  |  |  | 0.201 |
| Yes | 8 | 1.03 (1.00, 1.07) | 0.07 | 45.3 |  |
| No | 5 | 1.00 (0.95, 1.04) | 0.17 | 36.6 |  |
| Adjustment for alcohol consumption |  |  |  |  | 0.001 |
| Yes | 2 | 1.09 (1.04, 1.15) | 0.77 | 0.0 |  |
| No | 11 | 0.99 (0.96, 1.02) | 0.40 | 4.3 |  |
| Adjustment for family history of cancer |  |  |  |  | 0.204 |
| Yes | 5 | 1.00 (0.96, 1.04) | 0.95 | 0.0 |  |
| No | 8 | 1.04 (1.00, 1.08) | 0.01 | 62.0 |  |
| Study quality |  |  |  |  | 0.050 |
| NOS score ≥7 | 7 | 0.99 (0.95, 1.03) | 0.56 | 0.0 |  |
| NOS score <7 | 6 | 1.04 (1.01, 1.08) | 0.03 | 58.6 |  |
| Subgroup analyses for red and processed meat intake and risk of advanced prostate cancer | | | | | |
| US vs. Non-US |  |  |  |  | 0.039 |
| US | 8 | 1.07 (0.99, 1.15) | 0.01 | 60.0 |  |
| Non-US | 1 | 0.80 (0.61, 1.04) | . | . |  |
| Adjustment for energy intake |  |  |  |  | 0.079 |
| Yes | 7 | 1.06 (0.99, 1.14) | 0.005 | 67.9 |  |
| No | 2 | 0.81 (0.61, 1.09) | 0.93 | 0.0 |  |
| Adjustment for smoking |  |  |  |  | 0.234 |
| Yes | 7 | 1.05 (0.98, 1.13) | 0.002 | 70.5 |  |
| No | 2 | 0.82 (0.54, 1.23) | 0.86 | 0.0 |  |
| Adjustment for alcohol consumption |  |  |  |  | 0.000 |
| Yes | 2 | 1.24 (1.11, 1.39) | 0.44 | 0.0 |  |
| No | 7 | 0.92 (0.84, 1.01) | 0.55 | 0.0 |  |
| Adjustment for family history of cancer |  |  |  |  | 0.370 |
| Yes | 5 | 1.02 (0.93, 1.12) | 0.02 | 64.8 |  |
| No | 4 | 1.09 (0.97, 1.22) | 0.02 | 68.8 |  |
| Study quality |  |  |  |  | 0.617 |
| NOS score ≥7 | 5 | 1.07 (0.95, 1.22) | 0.02 | 63.0 |  |
| NOS score <7 | 4 | 1.03 (0.95, 1.13) | 0.01 | 72.1 |  |
| Subgroup analyses for total meat intake and risk of total prostate cancer | | | | | |
| US vs. Non-US |  |  |  |  | 0.812 |
| US | 12 | 1.03 (1.00, 1.06) | 0.10 | 36.4 |  |
| Non-US | 8 | 1.02 (0.96, 1.09) | 0.009 | 62.5 |  |
| Adjustment for energy intake |  |  |  |  | 0.006 |
| Yes | 11 | 1.01 (0.99, 1.04) | 0.08 | 40.3 |  |
| No | 9 | 1.14 (1.05, 1.24) | 0.16 | 31.7 |  |
| Adjustment for smoking |  |  |  |  | 0.287 |
| Yes | 11 | 1.04 (1.00, 1.08) | 0.03 | 47.5 |  |
| No | 9 | 1.01 (0.97, 1.05) | 0.04 | 49.4 |  |
| Adjustment for alcohol consumption |  |  |  |  | 0.001 |
| Yes | 3 | 1.10 (1.05, 1.15) | 0.64 | 0.0 |  |
| No | 17 | 1.00 (0.97, 1.03) | 0.08 | 33.4 |  |
| Adjustment for family history of cancer |  |  |  |  | 0.271 |
| Yes | 8 | 1.01 (0.98, 1.05) | 0.28 | 18.4 |  |
| No | 12 | 1.04 (1.00, 1.09) | 0.006 | 58.0 |  |
| Study quality |  |  |  |  | 0.012 |
| NOS score ≥7 | 8 | 0.99 (0.95, 1.03) | 0.67 | 0.0 |  |
| NOS score <7 | 12 | 1.06 (1.02, 1.09) | 0.009 | 55.8 |  |
| Subgroup analyses for total meat intake and risk of advanced prostate cancer | | | | | |
| US vs. Non-US |  |  |  |  | 0.077 |
| US | 10 | 1.11 (1.04, 1.18) | 0.005 | 62.1 |  |
| Non-US | 2 | 0.89 (0.70, 1.12) | 0.07 | 67.7 |  |
| Adjustment for energy intake |  |  |  |  | 0.111 |
| Yes | 8 | 1.06 (0.98, 1.14) | 0.008 | 63.3 |  |
| No | 4 | 1.19 (1.05, 1.35) | 0.03 | 64.2 |  |
| Adjustment for smoking |  |  |  |  | 0.547 |
| Yes | 9 | 1.09 (1.03, 1.17) | 0.001 | 70.7 |  |
| No | 3 | 0.99 (0.71, 1.37) | 0.32 | 12.3 |  |
| Adjustment for alcohol consumption |  |  |  |  | 0.005 |
| Yes | 2 | 1.24 (1.11, 1.39) | 0.44 | 0.0 |  |
| No | 10 | 1.02 (0.95, 1.10) | 0.01 | 57.9 |  |
| Adjustment for family history of cancer |  |  |  |  | 0.027 |
| Yes | 6 | 1.01 (0.92, 1.11) | 0.04 | 56.7 |  |
| No | 6 | 1.17 (1.07, 1.28) | 0.01 | 63.1 |  |
| Study quality |  |  |  |  | 0.782 |
| NOS score ≥7 | 5 | 1.07 (0.95, 1.22) | 0.02 | 63.0 |  |
| NOS score <7 | 7 | 1.10 (1.02, 1.18) | 0.004 | 68.6 |  |
| Subgroup analyses for total meat intake and risk of all outcomes of prostate cancer | | | | | |
| US vs. Non-US |  |  |  |  | 0.498 |
| US | 14 | 1.05 (1.02, 1.08) | 0.009 | 62.5 |  |
| Non-US | 8 | 1.02 (0.96, 1.09) | 0.003 | 58.6 |  |
| Adjustment for energy intake |  |  |  |  | <0.001 |
| Yes | 11 | 1.02 (0.99, 1.05) | 0.01 | 54.3 |  |
| No | 11 | 1.17 (1.09, 1.25) | 0.08 | 39.2 |  |
| Adjustment for smoking |  |  |  |  | 0.067 |
| Yes | 13 | 1.06 (1.03, 1.10) | 0.002 | 61.7 |  |
| No | 9 | 1.01 (0.97, 1.05) | 0.04 | 49.4 |  |
| Adjustment for alcohol consumption |  |  |  |  | 0.001 |
| Yes | 4 | 1.11 (1.06, 1.16) | 0.26 | 25.2 |  |
| No | 18 | 1.01 (0.98, 1.04) | 0.005 | 52.7 |  |
| Adjustment for family history of cancer |  |  |  |  | 0.125 |
| Yes | 9 | 1.02 (0.99, 1.06) | 0.05 | 46.9 |  |
| No | 13 | 1.07 (1.02, 1.11) | 0.001 | 63.7 |  |
| Study quality |  |  |  |  | 0.022 |
| NOS score ≥7 | 8 | 1.00 (0.96, 1.05) | 0.11 | 40.2 |  |
| NOS score <7 | 14 | 1.07 (1.03, 1.10) | 0.001 | 61.3 |  |

NOS: Newcastle-Ottawa Scale; US: United States; RR: relative risk; CI: Confidence Interval.
